# Intranasal application of a bifunctional pertactin-RTX fusion antigen elicits protection of mouse airway mucosa against *Bordetella pertussis* colonization

**DOI:** 10.1128/msphere.00959-24

**Published:** 2025-03-31

**Authors:** Carlos Espinosa-Vinals, Jana Holubova, Ondrej Stanek, Radim Osicka, Jiri Masin, Fresia Esther Arellano Herencia, Peter Sebo

**Affiliations:** 1 Laboratory of Molecular Biology of Bacterial Pathogens, Institute of Microbiology of the Czech Academy of Sciences, Prague, Czechia; University of Maryland School of Medicine, Baltimore, Maryland, USA

**Keywords:** *Bordetella pertussis*, adenylate cyclase toxin, pertactin, whooping cough, pertussis, protein folding, protection

## Abstract

The adenylate cyclase toxin (ACT, AC-Hly, or CyaA) plays a key role in airway infections by *Bordetella pertussis* and ablates the oxidative burst and opsonophagocytic capacity of sentinel phagocytes. CyaA fragments eliciting toxin-neutralizing antibodies are considered prime antigen candidates for improved acellular pertussis (aP) vaccines but their contribution to aP-mediated protection against *B. pertussis* infection awaits demonstration. We explored whether hybrid antigens inducing simultaneously CyaA-neutralizing and anti-Prn opsonizing antibody responses can enhance aP-elicited protection of mouse airways from infection. Fusion to the N-terminus of an RTX908 antigen derived from CyaA enabled an accelerated folding of the pertactin passenger domain (rPrn) in function of calcium loading of the RTX908 moiety and conferred on the rPrn-RTX908 fusion antigen a superior capacity to induce functional anti-Prn IgG antibodies. The rPrn-RTX908 fusion antigen also elicited CyaA neutralizing anti-RTX antibodies that relieved the toxin-imposed inhibition of oxidative burst and opsonophagocytic uptake of *B. pertussis* bacteria by HL-60 cells exposed to physiological concentrations of the CyaA toxin. Intranasal immunization of mice with the rPrn-RTX908 antigen admixed into a PT and FHA-based aP vaccine elicited specific sIgA responses in mucosal secretions (saliva) and conferred a significantly enhanced protection of mouse lung and nose mucosa against *B. pertussis* infection, yielding a significantly accelerated clearance of bacteria from the infected lungs within a single day from infection. These results demonstrate the added value of anti-CyaA antibodies elicited by intranasal application of the rPrn-RTX908 fusion antigen in the protection of the airway against *B. pertussis* infection.

**IMPORTANCE:**

Despite high vaccine coverage, unexpectedly massive whooping cough outbreaks are currently resurging in the most developed countries using the acellular pertussis (aP) vaccine. Accelerated development of improved aP vaccines, conferring a more complete and longer-lasting protection of the airway from *Bordetella pertussis* infection, is sorely needed. The highly immunosuppressive RTX adenylate cyclase toxin (CyaA) was proposed as a prime antigen candidate for inclusion into improved aP vaccines. We show here that a soluble RTX-derived antigen fused to the major opsonizing antibody target pertactin (rPrn-RTX908 hybrid) elicits opsonizing and toxin-neutralizing antibody responses that relieve the CyaA-imposed block of bactericidal opsonophagocytic uptake capacities of sentinel phagocytes. Intranasal immunization with the rPrn-RTX908 hybrid antigen then enables a significantly accelerated clearance of *B. pertussis* bacteria from mouse lungs and superior protection of mouse nasal mucosa from bacterial infection. These results unravel the added value of RTX antigen inclusion into the next generation of aP vaccines.

## INTRODUCTION

Pertussis, or whooping cough, is a highly contagious respiratory illness caused predominantly by *Bordetella pertussis* and less frequently by the human-adapted *B. parapertussis*_HU_ coccobacilli. Despite global pertussis vaccine coverage rates exceeding 83%, pertussis remains the least-controlled vaccine-preventable infectious disease. It is estimated that less than 1% of *B. pertussis* infections get diagnosed (1, 2) and about 20 million whooping cough cases, accounting for about 150,000 pertussis-related infant deaths, occur annually worldwide (3). Moreover, pertussis outbreaks resurged in a number of developed countries within 7–14 years from the introduction of acellular pertussis (aP) vaccines (4, 5) and since the fall of 2023 unexpectedly massive pertussis outbreaks are resurging throughout the highly aP-vaccinated populations of the EU and other high-income countries (6). Immunization and boosting with the aP vaccine also led to the emergence of *B. pertussis* mutants that do not produce pertactin (Prn), the key opsonizing and bactericidal antibody-inducing aP vaccine component (7–9). The current pertussis outbreaks and the circulation of Prn-deficient *B. pertussis* in highly aP-vaccinated populations reveal the urgent need for the development of the next generation of pertussis vaccines that would also restrict the transmission of the pathogen.

Among the promising antigen candidates for inclusion into such vaccines are the fragments of the repeats-in-toxin (RTX) family adenylate cyclase toxin (CyaA) of *B. pertussis* (10–16). CyaA preferentially targets sentinel phagocytes expressing the toxin receptor CD11b, the α_M_ subunit of the complement receptor 3 (CR3), known as the α_M_β_2_ integrin CD11b/CD18 (17–19). CyaA binds CR3 and delivers into phagocytes a cell-invasive adenylyl cyclase enzyme that catalyzes the uncontrolled conversion of cellular ATP into cAMP. The subversive signaling of accumulating cAMP then near-instantly blocks the uptake of complement-opsonized bacteria by phagocytes and annihilates their capacity to produce the bactericidal reactive oxygen species (ROS) (17, 19–22). Not surprisingly, hence, CyaA-neutralizing antibodies protect mice from a lethal *B. pertussis* infection and accelerate the clearance of bacteria from infected lungs (10–16, 23).

Phagocyte binding and cytotoxic activity, as well as the protective immunogenicity of CyaA, both critically depend on the proper structure of the CD11b-binding segment within the C-terminal RTX domain. This key functional structure of CyaA is formed in a process of calcium binding-dependent and series-templated C-to-N-terminal vectorial folding of the RTX domain, which yields a rigid assembly of 5 RTX β-rolls (blocks I–V) that are interspersed by β-structured linkers (L1–L4) (24–28). The RTX blocks I to III, with the linkers L1 and L2, constitute the CD11b-binding structure that comprises the protective conformational epitopes of CyaA (17, 26, 27, 29). Thanks to a mechanistic insight into the RTX domain folding process, we and others could generate folded and soluble CR3-binding fragments of the RTX domain that can elicit CyaA-neutralizing antibodies capable of blocking toxin binding to phagocytes (26, 30).

**Fig 1:**
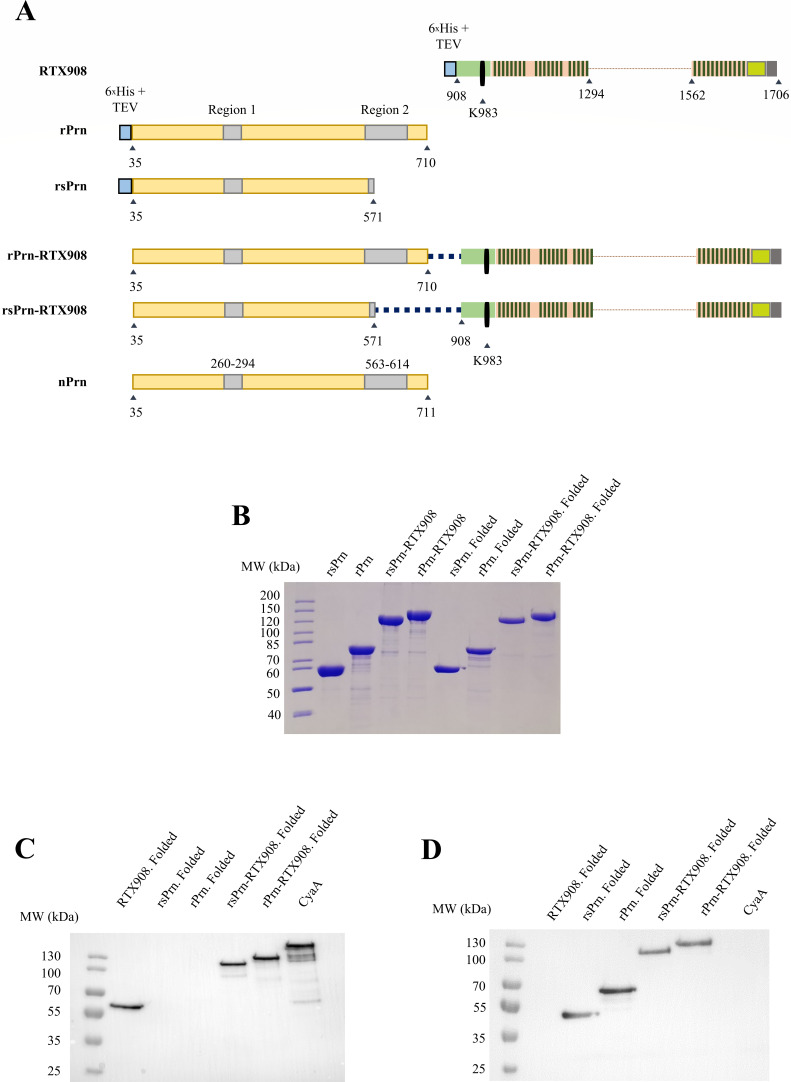
Used antigens. (**A**) Schematic depiction of the pertactin-derived constructs rPrn and rsPrn, and their fusions with the RTX908 protein moiety (30). The 6×His affinity tag followed by a TEV protease cleavage site is indicated as a blue rectangle. The immunogenic regions 1 and 2 of pertactin are indicated by gray rectangles. The acylated domain of CyaA is in green and the RTX blocks I, II, and III/V of RTX908 are given as blocks of dark green bars. Numbers below the bars indicate residues of Prn and CyaA comprised in the constructs. (**B**) Coomassie Blue stained SDS-PAGE gel (7.5%) of the purified proteins used for refolding and immunization experiments. On-column lipopolysaccharide (LPS) removal was conducted in an 8 M urea-containing buffer as previously described (38). Folding of proteins (samples labeled as Folded) was performed according to a modified protocol of Hijnen et al. (34), using a buffer containing 200 mM L-Arg, 3 mM CaCl_2_, 100 mM NaCl, protease inhibitors cocktail, and 50 mM Tris-HCl, pH 8. The proteins were concentrated by ultrafiltration on a membrane with a 30 kDa cutoff. (**C and D**) Western blots of the folded proteins. 0.5 µg of protein per lane was separated on a 7.5% SDS-PAGE, transferred onto a PVDF membrane, and detected using either a polyclonal mouse anti-CyaA serum (**C**) or the anti-Prn monoclonal antibody PeM72 targeting the linear epitope in Region 1 of Prn (**D**).

In this study, we constructed the proteins rPrn and rsPrn (Fig. 1A) that comprise the passenger domain of aP vaccine antigen Prn, including or not the hypervariable loop Region 2, respectively. We fused the CyaA-derived RTX908 fragment antigen to the C-terminal end of rPrn and rsPrn proteins to generate hybrid antigens (rsPrn-RTX908 and rPrn-RTX908; see Fig. 1A) capable to elicit functional antibodies against both Prn and CyaA. We reasoned that if the Prn and RTX908 moieties were fused in a correct folding phase, the C-to-N-terminal vectorial folding signal triggered by Ca^2+^ binding to the RTX908 moiety might be transmitted onto the attached N-terminal Prn passenger moiety and might drive its C-to-N-terminus vectorial folding into the antigenic right-handed β-helix structure of folded Prn antigen (31–33). We showed that two differently sized Prn-RTX908 hybrid antigens can simultaneously induce both opsonizing anti-Prn and CyaA toxin-neutralizing antibodies. Such bispecific sera enabled enhanced opsonophagocytic uptake of *B. pertussis* bacteria by differentiated HL-60 (dHL-60) neutrophils and relieved the block of bactericidal ROS production by phagocytes exposed to added CyaA toxin. Moreover, intranasal immunization of mice with the rPrn-RTX908 fusion antigen admixed into an aP vaccine importantly enhanced the immune protection of mouse lungs against infection by *B. pertussis* and significantly reduced nasal mucosa colonization.

## RESULTS

### Prn fused to an RTX fragment folds faster and displays immunodominant epitopes of native pertactin

The recombinant Prn antigen extracted in denaturing buffers from *Escherichia coli* inclusion bodies exhibits very slow folding kinetics (34, 35). We thus hypothesized that the rapid Ca^2+^-driven C-to-N-terminal vectorial folding of a β-roll-structured RTX domain fused to the C-terminal end of Prn could also promote a rapid calcium-induced folding of the Prn moiety into a β-solenoid structure, providing that the two β-structured moieties were fused in a proper folding phase (30). From several previously characterized RTX domain fragments (i.e. RTX770, RTX908, and RTX1008), which fold in the presence of Ca^2+^ ions and competitively bind the CR3 receptor (Fig. S1), we chose the RTX908 construct as an optimal Prn fusion partner moiety in terms of size, thermal stability, and CR3 binding capacity (30). The used RTX908 fragment lacks the residues 1,295 to 1,561 of CyaA, but still comprises a portion of the N-terminal cap of the RTX domain bearing the CyaC-acylated K983 residue (Fig. 1A) and forms a properly folded CR3-binding structure (17, 30, 36).

Therefore, two differently sized recombinant Prn moieties were tested in the fusion constructs (Fig. 1A). The shorter Prn (rsPrn) moiety (residues 35–571) contained the immunodominant region 1 (residues 260–294) and corresponds to the Prn segment of the known 3D structure (PDB 1DAB) (33). The longer rPrn protein (residues 35–710) comprised the P.69 segment included in the aP vaccine and contains also the presumably flexible unstructured loop, which according to the Alphafold prediction (37) harbors the immunodominant region 2 (residues 563–614) followed by a predicted three β-strand extension coming already from the autotransporter β-barrel moiety (Fig. S2 and S4). The rsPrn-RTX908 and rPrn-RTX908 proteins were produced in *E. coli* BL21/pMM100(*lacI^q^*), extracted with 8 M urea from washed inclusion bodies and purified close to homogeneity under denaturing conditions (Fig. 1B), including an on-column LPS removal step (38), allowing to obtain proteins with very low endotoxin content (Table 1). Protein identity was then verified by Western blots with a polyclonal mouse serum recognizing CyaA (Fig. 1C) and the Prn-specific monoclonal antibody PeM72 (Fig. 1D).

**TABLE 1:** Protein molecular weight and endotoxin content^*a*^

Protein	MW (kDa)	Endotoxin (EU/mg of protein)
rsPrn *LPS−*	57.7	152
rsPrn-RTX908 *LPS−*	111.3	55
rPrn *LPS−*	71.9	42
rPrn-RTX908 *LPS−*	125.7	56
RTX908 *LPS−*	57.6	38
nPrn *LOS−*	72.1	200
nPrn *LOS+*	72.1	4,667
nPrn (SII)	72.1	20

^*a*^ *LPS*−, samples depleted of *Escherichia coli* LPS through washing with Triton X114; *LOS*−, samples depleted of *Bordetella pertussis* LOS through washing with Triton X114, following the method described by Stanek et al. (38); nPrn (SII), native pertactin kindly shared by Serum Institute of India; EU, endotoxin units.

**Fig 2:**
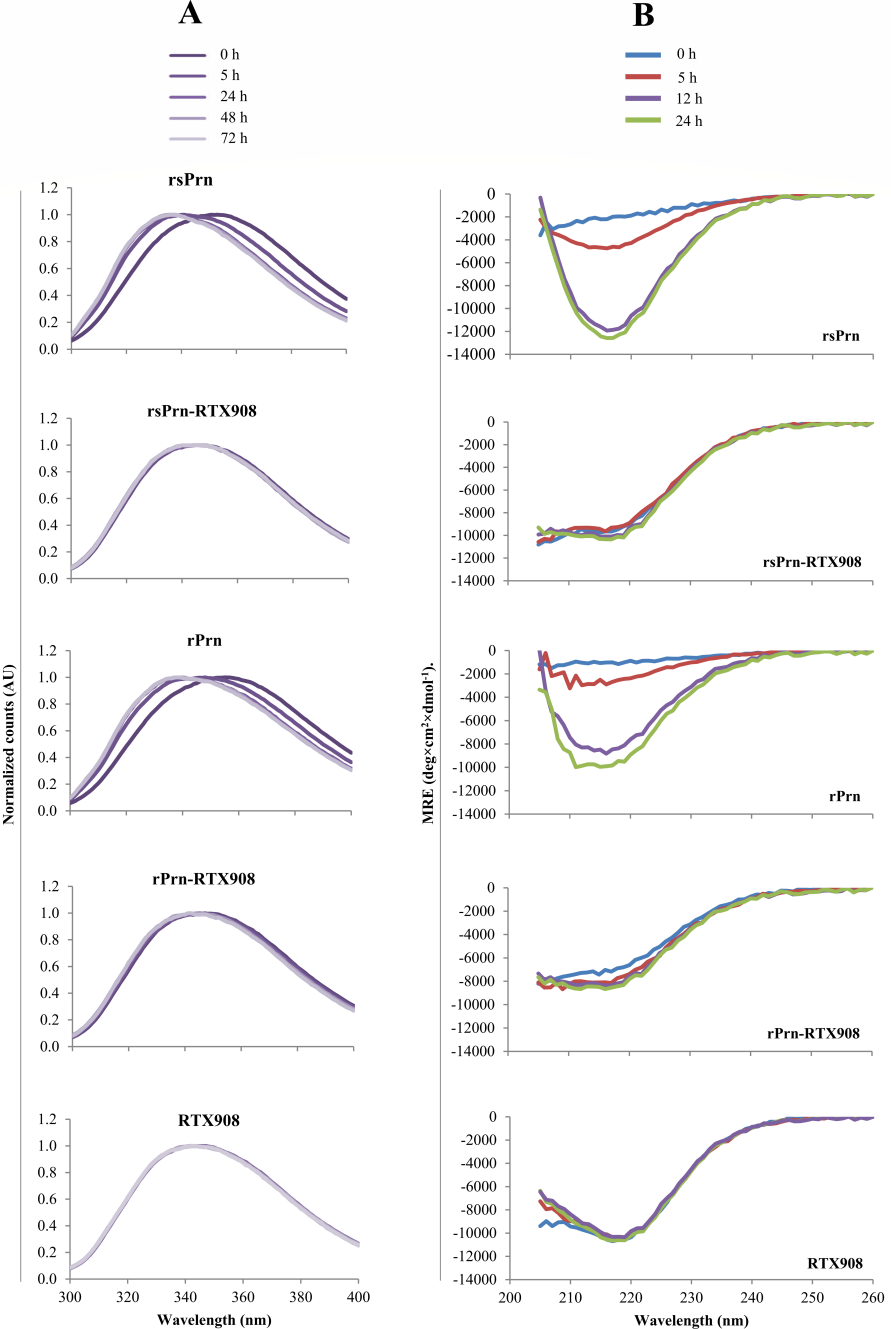
Calcium loading-driven folding of the RTX908 moiety enables importantly accelerated folding of the N-terminally fused rPrn moiety. (**A**) Folding kinetics of the used antigenic proteins as monitored by fluorescence spectroscopy. The proteins were refolded upon direct >60-fold dilution of the 8 M urea-containing stock solutions to a 0.2 mg/mL protein final concentration in urea-free buffer containing 2 mM Ca^2+^ and incubated at 4°C. The shift in tryptophan fluorescence from 350 to 330 nm, measured as the intrinsic fluorescence of the tryptophan residues, was recorded at time periods indicated by the shading of the spectroscopic curves at time points 0 h, 5 h, 24 h, 48 h, and 72 h. The curves represent the average of the two experiments. (**B**) Folding kinetics of the proteins monitored by circular dichroism spectroscopy. The protein stocks were diluted to 0.2 mg/mL as above and incubated for 0 h, 5 h, 12 h, and 24 h at 4°C. Circular dichroism signal was collected in the range of 205–260 nm at room temperature using 0.1 cm path-length quartz cuvettes. The measured ellipticities were converted into mean residue ellipticity (MRE) and expressed in deg × cm^2^ × dmol^−1^. The curves correspond to the average of two experiments conducted in duplicate.

To analyze the folding kinetics of the free and RTX908-fused rsPrn and rPrn moieties, the proteins were refolded from a denaturing 8 M urea solution by direct >60 fold dilution into urea-free buffer containing 2 mM Ca^2+^ and the shift in tryptophan fluorescence from 350 to 330 nm was monitored over time. As shown in Fig. 2A, the Prn-RTX908 fusion proteins rapidly folded upon simple dilution into calcium-containing buffer, as judged from the overlap of curves recorded at different time points, where the instantly stable tryptophan fluorescence did not change over time of incubation at 4°C. By contrast, the unfused rsPrn and rPrn proteins underwent slow folding that took more than 5 hours to reach a state at which the tryptophan residues got buried inside the molecule and became shielded from the aqueous environment. This difference in folding kinetics of free and RTX908-fused Prn moieties was further corroborated by circular dichroism spectroscopy. Indeed, CD spectra corresponding to a folded state of the rsPrn-RTX908 and rPrn-RTX908 fusion proteins were observed directly upon dilution from the denaturing 8 M urea buffer into the buffer containing 2 mM Ca^2+^, whereas completion of folding of the free rsPrn and rPrn proteins took over 12 hours (Fig. 2B).

**Fig 3:**
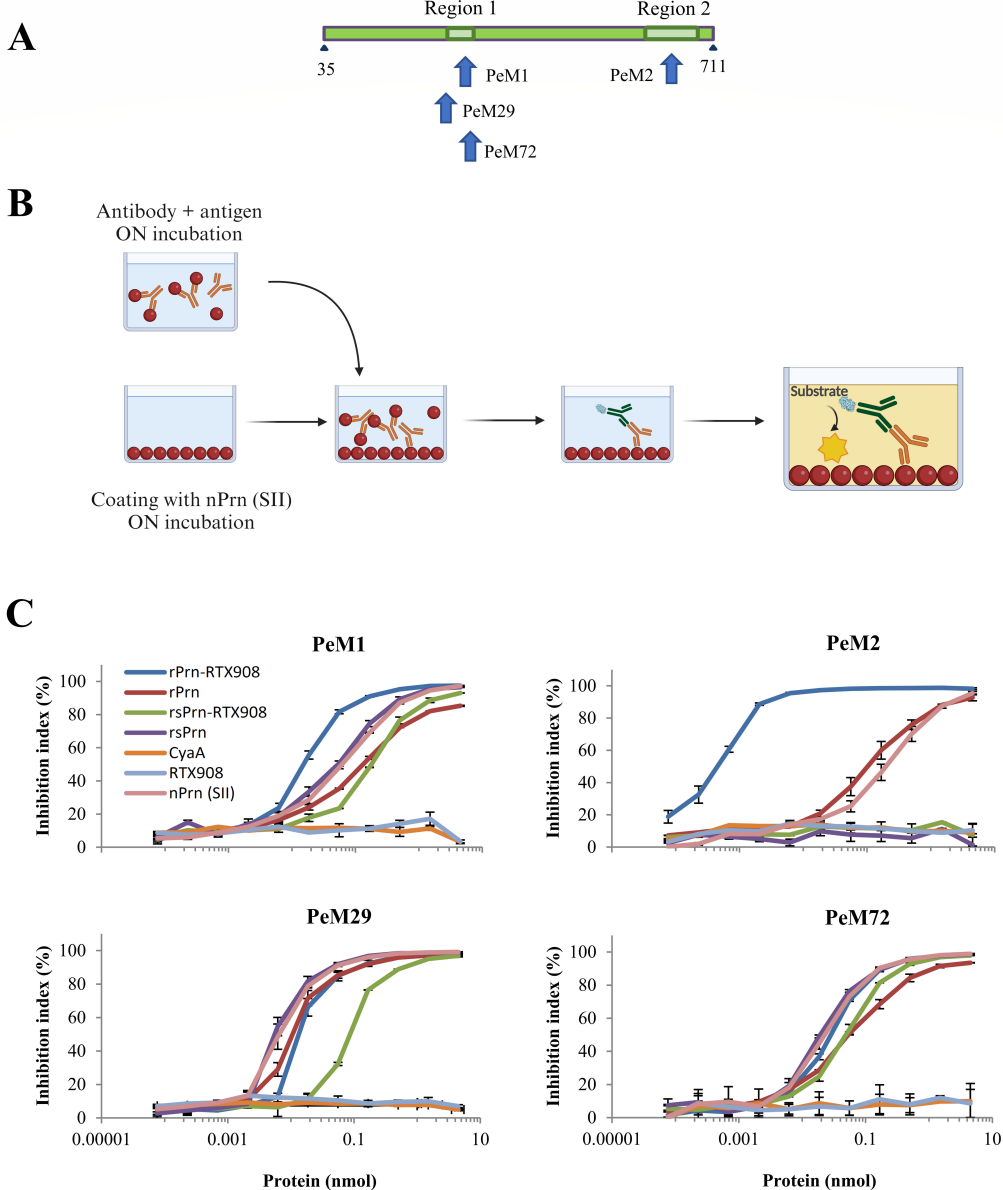
Calcium loading-driven folding of the RTX908 moiety enables enhanced presentation of the immunodominant Region 1 and Region 2 epitopes of the fused rPrn moiety. (**A**) Schematic depiction of the passenger domain of Prn and the location of the immunodominant Prn epitopes recognized by the monoclonal antibodies PeM1, PeM2, PeM29, and PeM72 (35, 39). (**B**) Scheme of the inhibition ELISA employed to evaluate the antigenicity of the recombinant Prn protein variants. The specific anti-Prn antibody (PeM1, PeM2, PeM29, or PeM72), at a suitable concentration from the linear dose-response range determined in preliminary experiments, was incubated with increasing concentrations of the respective antigen for 16 h at 4°C. The reactions were next transferred to nPrn-coated plates to capture the remaining free antibody molecules that did not bind to the tested antigen. The plates were washed repeatedly, and an anti-mouse IgG antibody conjugated to horseradish peroxidase was added to detect the amounts of mouse IgG captured on the plates, using TMB/H_2_O_2_ as chromogenic substrate, and the absorbance at 450 nm was read. (**C**) Recognition of the Region 1 and Region 2 epitopes of free and RTX908-fused recombinant pertactin moieties by anti-Prn mAbs. Increasing concentrations of the tested proteins were incubated overnight at 4°C with the monoclonal antibodies recognizing conformational (PeM1, PeM2, and PeM29) and linear (PeM72) epitopes of pertactin (40). The curves represent the average of two independent measurements of the inhibition index calculated for each concentration point. The error bars indicate the standard deviation. CyaA and free RTX908 were used as negative control antigens for background determination.

The above results suggested proper folding of the Prn moieties when fused to RTX908. To verify that, we used a set of Prn-specific monoclonal antibodies recognizing conformational (i.e. PeM1, PeM2, PeM29) and linear (PeM72) epitopes of Prn (35, 39, 40) to probe the formation and presentation of these epitopes by the free and RTX908-fused rsPrn and rPrn protein moieties (Fig. 3A). To enable the comparisons, both free and fused Prn proteins were refolded from 8 M urea buffer using the same modified protocol of Hijnen et al*.* (34). Following a 17.5-fold dilution of protein stocks into a buffer containing 200 mM L-Arg and 2 mM Ca^2+^, the residual urea was removed by ultrafiltration in the same buffer, and folding of the proteins was allowed to complete over 24 hours at 4°C. Such refolded rsPrn and rPrn proteins and their RTX908-fused variants were then characterized for the presentation of the epitopes in an inhibition ELISA setup (Fig. 3B). Native nPrn purified from *B. pertussis* cultures (Prn P.69 antigen) was coated onto ELISA plate wells and the folded recombinant rsPrn and rPrn proteins and their RTX908 fusion versions were added as competitors to inhibit the binding of the anti-Prn antibodies to the coated nPrn antigen. Since the epitope for PeM2 was absent in the truncated rsPrn protein or its rsPrn-RTX908 fusion, it was not recognized by PeM2 (Fig. 3C). By difference, the rPrn-RTX908 fusion was recognized by both the PeM1 and PeM2 mAbs at importantly lower concentrations than the rsPrn and rPrn proteins, or the native nPrn protein purified from *B. pertussis* cells. This suggests that the region 1 and region 2 loops of Prn, involved in the induction of opsonizing protective anti-Prn antibodies (34, 41), were well presented by the folded rPrn-RTX908 fusion protein. In particular, the conformational epitope recognized by the PeM2 antibody appeared to be much better structured and recognized in the rPrn-RTX908 fusion context than in the free native Prn protein, where region 2 appears to form a flexible loop that could not be resolved in the crystal structure of Prn (c.f. Fig. S2 to S4). The rPrn protein or its fusion to RTX908 was then recognized by the other two mAbs, PeM29 and PeM72 at least as well as the native nPrn protein, showing that even the free unfused rPrn folded properly.

### The rPrn-RTX908 fusion antigen elicits opsonizing and CyaA-neutralizing antibodies that enable oxidative burst and opsonophagocytic uptake of *B. pertussis* by neutrophils exposed to CyaA toxin

**Fig 4:**
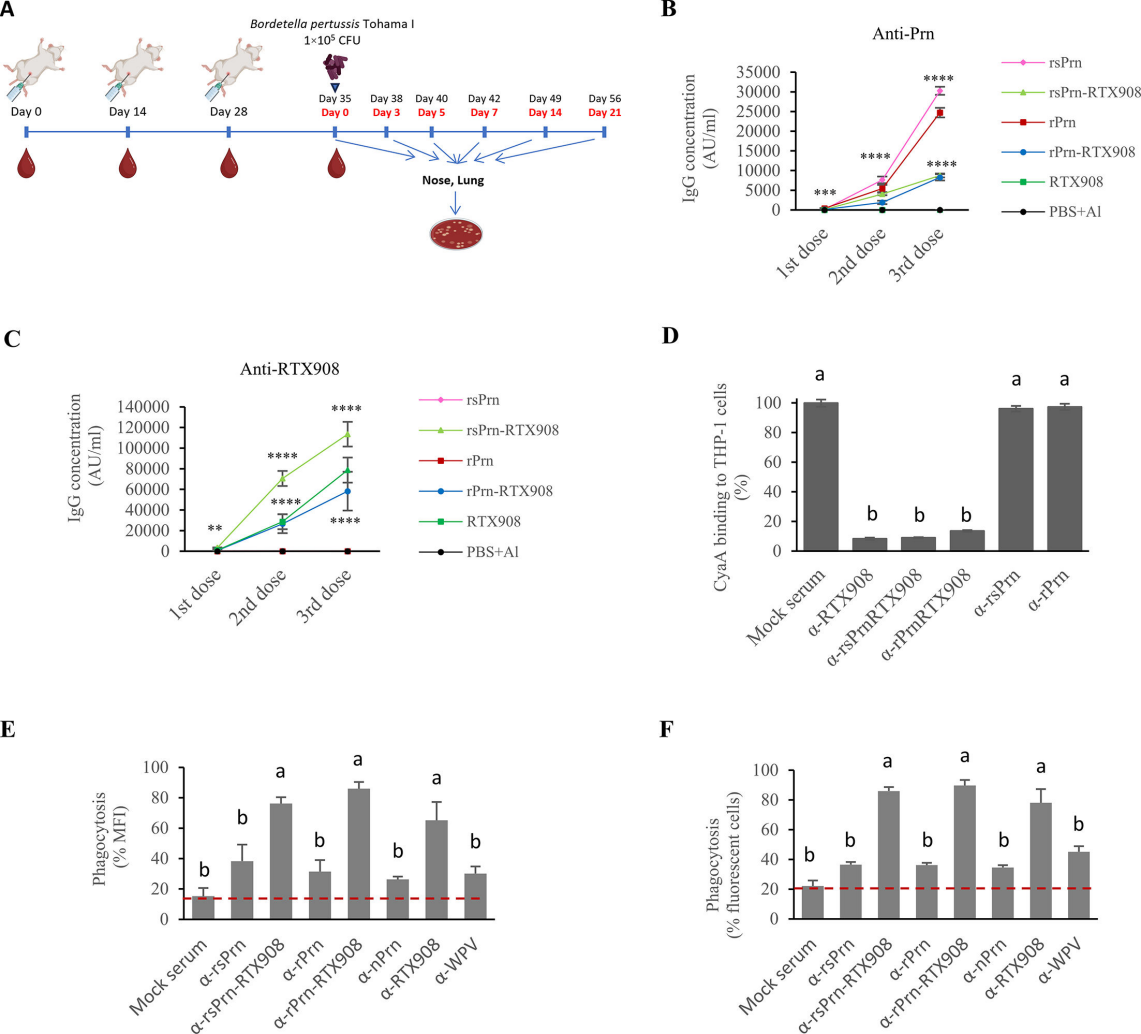
rPrn-RTX908 antigens elicit both opsonizing and CyaA toxin-neutralizing serum antibodies that enable opsonophagocytic uptake of *B. pertussis* bacteria by neutrophils in the presence of CyaA and enhance the protection of mouse lung against *B. pertussis* infection. (A) Scheme of the immunization and infection experiment. BALB/cByJ mice (*n* = 15) were intraperitoneally vaccinated with three doses of 31.8 pmol of the individual proteins at days 0, 14, and 28. Sera were collected on day 0 (pre-immune), day 14 (1st dose), day 28 (2nd dose), and day 35 (3rd dose). The animals were next infected with 50 µL of 1 × 10^5^ CFU of *B. pertussis* WT administered intranasally. At indicated times, the animals were sacrificed, and the bacterial load in the lungs and nasal cavities was determined by plating dilutions of lung and nasal tissue homogenates on Bordet Gengou blood agar plates for CFU counting after 5 days of cultivation at 37°C. (B, C) The relative concentrations of antigen-specific serum IgG were determined using arbitrary standard curves. Native pertactin (B) or purified RTX908 protein (C) were coated at 3 µg/mL onto Nunc Maxisorp ELISA plates. A standard curve for Prn-specific IgG quantitation was constructed using the monoclonal antibody (PeM72) and a polyclonal anti-CyaA serum was used for the standard curve of anti-RTX908 antibodies. The relative concentrations of anti-Prn IgG (B) and anti-RTX908 IgG (C) in the sera were determined in the linear dose-response range of the titration curves (AU/mL). For anti-pertactin antibodies, a relative concentration of 75 AU/mL was equivalent to 1 µg/mL, based on a calibration curve constructed using a monoclonal antibody with a known concentration. Geometric means of three independent determinations performed in duplicates are shown. The error bars indicate the error of the mean. The antibody concentrations of the experimental groups were compared to the levels of the negative group (PBS + Alum) using a nonparametric Mann-Whitney test. **, *P* < 0.01; ***, *P* < 0.001; ****, *P* < 0.0001. (D–F) CyaA toxin-neutralizing serum IgG antibodies elicited by rPrn-RTX908 antigenic hybrid enable opsonophagocytic uptake of *B. pertussis* bacteria by activated HL-60 cells exposed to inhibitory CyaA toxin concentration. (D) CyaA toxin neutralization was determined as inhibition of CyaA binding to its CD11b/CD18 receptor (CR3) on THP-1 cells. CyaA at 1 µg/mL was preincubated in D-MEM medium with 1:50 diluted mouse sera for 30 min at 4°C and added to THP-1 cells (1 × 10^6^) for an additional 30 min of incubation at 4°C. Unbound CyaA was removed by three cell washes and the adenylate cyclase enzyme activity of the cell-bound toxin was determined. CyaA binding was expressed as the percentage of toxin bound to THP-1 cells, where 100% corresponds to CyaA binding in the presence of mock serum. The data represent the mean ± S.D. of three independent experiments performed in duplicates. Statistical significance was assessed by the Kruskal-Wallis test followed by *post-hoc* Dunn´s multiple comparison test. Statistical differences among experimental groups are represented by letters (groups with differing letters do differ significantly). (E and F) Opsonophagocytic uptake of *B. pertussis* bacteria is inhibited by CyaA toxin (30 ng/mL) unless the toxin activity is blocked by toxin-neutralizing anti-RTX908 antibodies. Fluorescent mScarlet-expressing bacteria were opsonized with heat-inactivated mouse polyclonal sera (dilution 1:10) and baby rabbit serum serving as complement source (dilution 1:10) for 15 min at 37°C. CyaA was added to a final concentration of 30 ng/mL before DMF-differentiated HL-60 cells were added to reach an MOI of 20:1. After incubation for 45 min at 37°C in the absence or presence of 30 ng/mL of CyaA, the uptake of opsonized bacteria (E, F) was determined by flow cytometry. The results were expressed as the percentage of the specific parameter (MFI, percentage of HL-60 neutrophil cells containing mScarlet fluorescent bacteria) taking the respective CyaA-untreated wells as 100%. The graphs illustrate the geometric mean of the fluorescence of dHL-60 cells resulting from the phagocytosis of mScarlet-expressing bacteria (E) and the percentage of bacteria-containing differentiated HL-60 cells (F). Left panels; *, *P* < 0.05; **, *P* < 0.01; ***, *P* < 0.001; ****, *P* < 0.0001. Right panels, statistical differences are shown by different letters.

Prn is a major protective antigen of *B. pertussis* and the levels of opsonizing anti-Prn antibodies correlate with protection against pertussis disease (9, 42–45). We thus compared the immunogenicity of the rsPrn and rPrn proteins with that of the Prn-RTX908 fusions, performing intraperitoneal immunization of mice by three subsequent equimolar doses (31.8 pmol/dose) of the folded and alum-adsorbed recombinant proteins in 14-day intervals (Fig. 4A). As shown in Fig. 4B all recombinant proteins induced high levels of Prn-specific antibodies, which reached >5,000 AU/mL of serum IgG binding the rs-Prn-RTX908 and rPrn-RTX908 fusions and >25,000 AU/mL of IgG binding the free rsPrn and rPrn proteins, respectively.

**Fig 5:**
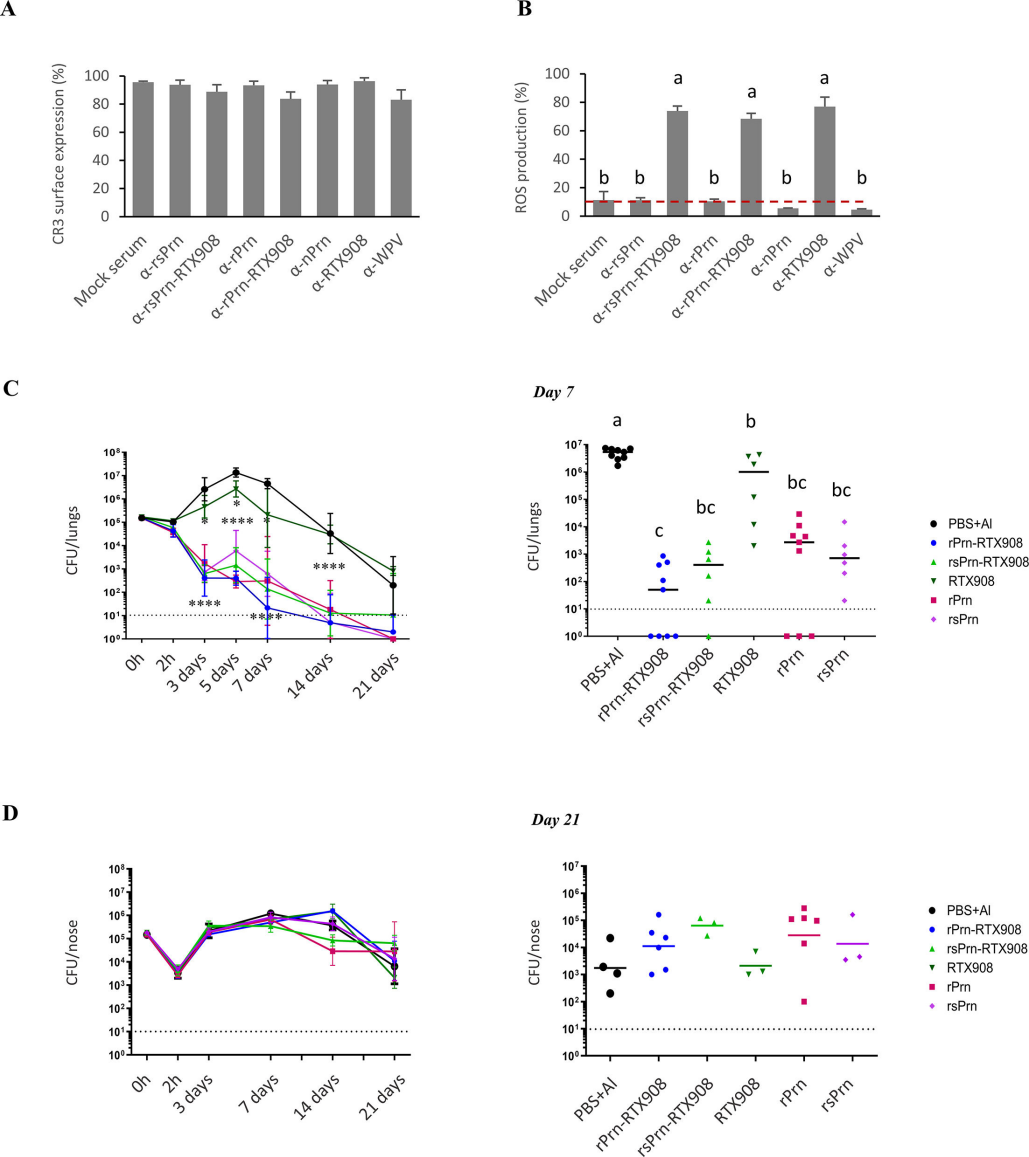
rPrn-RTX908 antigens elicit both opsonizing and CyaA toxin-neutralizing serum antibodies that enable opsonophagocytic uptake of *B. pertussis* bacteria by neutrophils in the presence of CyaA and enhance the protection of mouse lung against *B. pertussis* infection. Surface expression of the CR3 (CD11b/CD18) integrin (A) was determined using the anti-CD11b monoclonal antibody OKM1 conjugated to Dyomics 488. Differentiated HL-60 cells were included as a control of basal expression of CR3 in the absence of stimuli. Kruskal-Wallis test followed by Dunn´s multiple comparison test. Different letters indicate statistical differences. (B) Neutralization of CyaA toxin by anti-RTX908 antibodies enables reactive oxygen species (ROS) production by differentiated HL-60 cells (dHL-60) phagocytosing opsonized *B. pertussis* bacteria. dHL-60 cells were loaded with 150 µM luminol for 15 min at 37°C and incubated with opsonized *B. pertussis* bacteria in the presence and absence of CyaA at 30 ng/mL. The luminescence was continuously collected for 3 h at 90 second intervals. The bars indicate the geometric means of the percentage calculated from the area under the curve (AUC) of three independent experiments performed in triplicates (*n* = 9). The conditions incubated without CyaA were considered as the 100% to calculate the percentage of ROS production. Kruskal-Wallis test followed by *post- hoc* Dunn’s multiple comparison test was used for statistical analysis. Different letters indicate statistical differences. (C, D) Immune responses elicited by intraperitoneal immunization with the rPrn-RTX908 fusion antigen confer higher protection of mouse lungs from *B. pertussis* infection than the immunization with folded free rPrn antigen alone. Animals were intraperitoneally immunized with 31.8 pmol of the indicated recombinant antigens in three doses and were next intranasally infected with 50 µL of 1 × 10^5^ CFU of *B. pertussis* Tohama I suspensions. Bacterial loads in the lungs (C) and noses (D) were determined. Data points are geometrical means of the colony forming units determined in homogenates of tissue of the indicated numbers of animals per time point and represent the sum of data from three independent experiments. Statistical comparison to the mock-immunized control group (PBS + Al) is indicated (left panels). The right panels illustrate the bacterial load in the lungs (C, right panel) and nasal cavity (D, right panel) for individual animals on day 7 (C, lungs) and 21 (D, noses) post-infection, respectively. The horizontal bars in the right panels represent the geometric means calculated for each experimental group. The dashed lines indicate the detection limit of the assay. Compared using Kruskal-Wallis test and the *post-hoc* Dunn's multiple comparison test. Left panels; *, *P* < 0.05; **, *P* < 0.01; ***, *P* < 0.001; ****, *P* < 0.0001. Right panels, statistical differences are shown by different letters.

Importantly, the calcium-loaded rsPrn-RTX908 and rPrn-RTX908 fusion proteins elicited high levels of RTX-specific antibodies as the free RTX908 protein (Fig. 4C), and the sera raised against the fusion antigens blocked equally well the CR3-mediated binding of CyaA to human THP-1 monocyte/macrophage cells (Fig. 4D). Therefore, we next examined if such sera could relieve the inhibition of opsonophagocytic uptake of *B. pertussis* bacteria imposed on activated dHL-60 neutrophils by cAMP signaling resulting from CyaA toxin action (46, 47). In the absence of added CyaA toxin, the dHL-60 cells effectively phagocytosed complement-opsonized *B. pertussis* bacteria preincubated with mock sera supplemented with 10% baby rabbit complement, or the bacteria opsonized with the anti-Prn sera supplemented with 10% baby rabbit complement. By contrast, exposure to 30 ng/mL of CyaA resulted in significant inhibition of uptake of bacteria opsonized by complement only, or by complement plus monospecific anti-Prn sera raised against rsPrn or rPrn (Fig. 4E and F). Only the bispecific sera, which contained both anti-Prn opsonizing antibodies and CyaA-neutralizing antibodies induced by immunization with the rsPrn-RTX908 or rPrn-RTX908 fusion proteins, enabled a high level of opsonophagocytic uptake of bacteria even in the presence of 30 ng/mL of CyaA. Exposure of dHL-60 cells to CyaA toxin in the absence of CyaA-neutralizing antibodies did not decrease the surface levels of CR3 on dHL-60 cells (Fig. 5A), but CyaA action strongly suppressed the capacity of the dHL-60 cells to undergo oxidative burst and produce ROS. This was relieved when the CyaA-neutralizing antibodies were present in the sera (Fig. 5B). Hence, the sera raised against the fusion antigens were functionally superior in enabling opsonophagocytic uptake of *B. pertussis* bacteria and oxidative burst of dHL-60 neutrophils in the presence of CyaA toxin added at concentrations expected to occur on the infected mucosa *in vivo* (48).

### Simultaneous induction of anti-Prn and CyaA-neutralizing antibodies confers enhanced protection of mouse lungs from *B. pertussis* infection

We next examined whether the capacity to induce opsonizing (anti-Prn) and CyaA toxin-neutralizing (anti-RTX) antibodies in parallel confers an enhanced capacity on the Prn-RTX908 fusion antigen to elicit protective immunity against infection of the airways by *B. pertussis*. As shown in Fig. 5C, intraperitoneal immunization with equimolar amounts (31.8 pmol/dose) of either of the recombinant Prn protein variants and with either of its Prn-RTX908 fusions (c.f. Fig. 4A), conferred a high level of protection of mouse lungs against infection by 1 × 10^5^ CFU of *B. pertussis* Tohama I bacteria applied intranasally in 50 µL of suspension, hence in the *B. pertussis* mouse lung clearance model that predicts the clinical efficacy of licensed pertussis vaccines (49). Compared to mock-vaccinated mice that received only buffer with alum, and relative to the mice group immunized by only the RTX908 antigen, no initial proliferation of the administered bacteria in the lungs of mice immunized with the Prn protein variants was observed. The viable culturable bacterial counts (CFU) in lung homogenates of animals immunized with Prn or the Prn-RTX908 antigens declined steeply over the first 3 days after bacterial challenge (Fig. 5C). The rPrn-RTX908 fusion reproducibly granted the fastest decrease in *B. pertussis* CFU counts in the infected lungs in three experiments that yielded the same outcome, where four out of nine infected animals had completely cleared the infection by day 7 and five out of nine animals had only low detectable viable bacterial counts (~10^2^–10^3^ CFU) in the lungs 7 days after infection (Fig. 5C, right panel). This suggested an added value of the synergic action of CyaA-neutralizing antibodies with the anti-Prn-opsonizing antibodies in clearing *B. pertussis* bacteria from the infected mouse lungs. Moreover, the protective immunogenicity of the recombinant LPS-free rPrn and rPrn-RTX908 antigens was fully comparable to that of the vaccine-grade native nPrn antigen purified from *B. pertussis* cells, irrespective of whether this was depleted of residual *B. pertussis* LOS contamination or not (Fig. S5). These data confirm previous observations that anti-Prn IgG antibodies alone confer a very high level of mouse lung protection from *B. pertussis* infection (16, 39, 44, 50) and indicate that this protection can still be enhanced by a parallel induction of CyaA-neutralizing antibodies that alleviate CyaA-mediated inhibition of opsonophagocytic killing and clearance of the bacteria by myeloid phagocytes.

### Intranasal immunization with the rPrn-RTX908 antigen elicits superior protective immunity in the lungs and significantly restricts nasal mucosa colonization by *B. pertussis*

In line with previous reports (14, 51, 52), none of the intraperitoneally administered and IgG-inducing alum-adjuvanted protein vaccines conferred any significant protection of mouse nasal cavity mucosa from a long-lasting *B. pertussis* infection following inoculation with 10^5^ CFU of the bacterium (Fig. 5D). Indeed, 21 days after infection the bacterial loads in the nasal cavities of infected mice were similar for all the experimental groups, irrespective of whether the mice were immunized with the recombinant vaccines or not (Fig. 5D).

**Fig 6:**
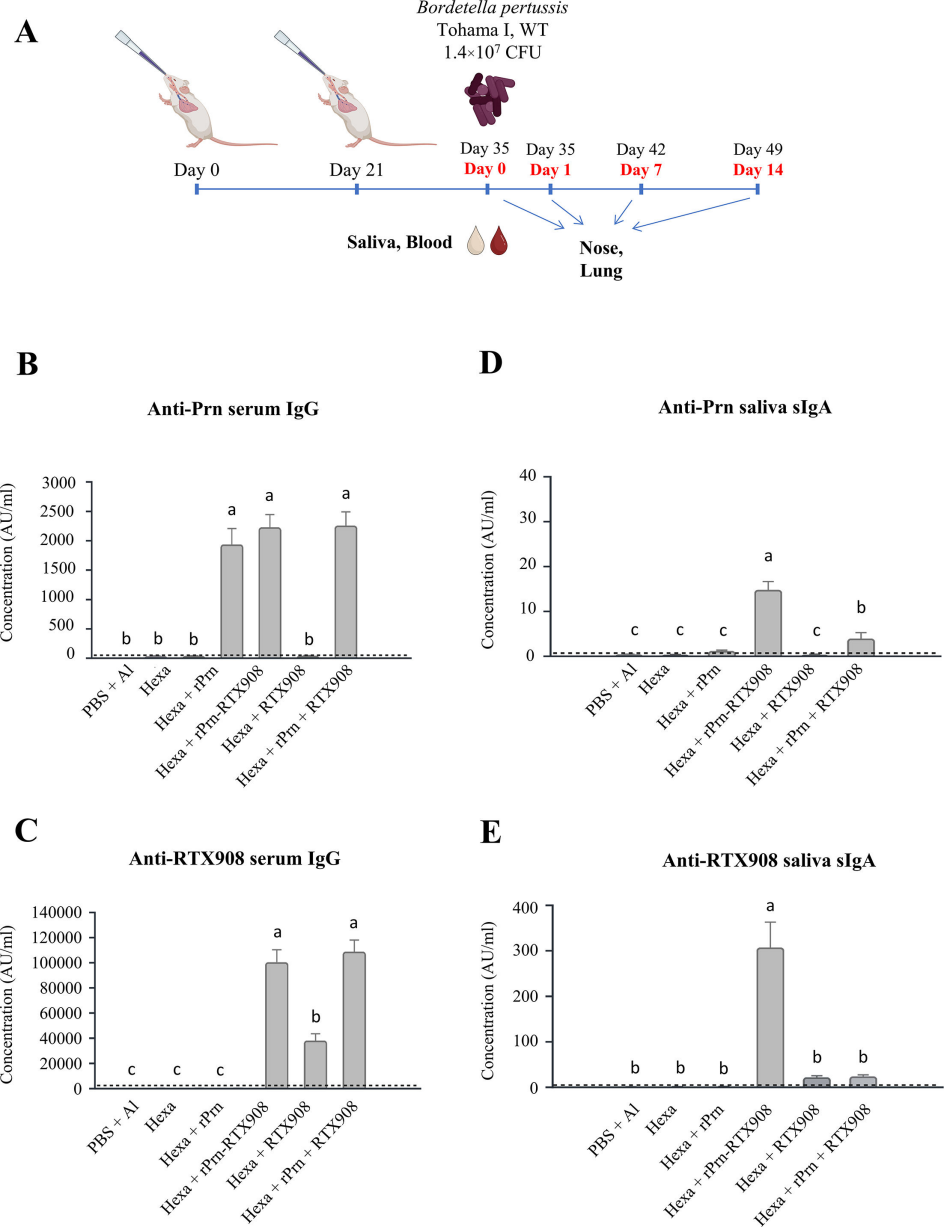
The rPrn-RTX908 fusion antigen is a potent mucosal immunogen that importantly enhances the protective efficacy of an intranasally applied 1/20 reduced dose of the pediatric pertussis vaccine and overrides its blunting effect on nasal cavity protection from *B. pertussis* infection. (A) Schematic depiction of the immunization experiment. Female BALB/cByJ mice were immunized twice at 21 days apart by intranasal application of 2 × 15 µL of 1/20 HD of pediatric hexavaccine (Hexacima) containing the PT and FHA pertussis antigens, which was supplemented with 14 pmol of the indicated antigens or their combination. Two weeks after the second vaccine dose, blood was sampled by retroorbital puncture, and saliva samples were obtained for serology. The animals were next infected by intranasal application of 1.4 × 10^7^ CFU of *B. pertussis* WT bacteria in 20 µL of suspension (10 µL per nostril). Bacterial load in lungs and nasal cavity tissue was determined at indicated time points. (B) Intranasal application of the recombinant antigens triggers potent anti-Prn serum antibody responses. Total anti-Prn serum IgG concentration (AU/mL) elicited by intranasal immunization with the acellular vaccine formulations were determined by PeM72 mAb-calibrated ELISA assay. (C) Relative concentration (AU/mL) of total anti-RTX908 serum IgG antibodies induced by intranasal immunization 1/20 HD hexavaccine formulations supplemented with indicated antigens (14 pmol). (D, E) Anti-Prn (D)- and anti-RTX908 (E)-specific antibody concentrations determined in saliva from uninfected animals (*n* = 6) immunized with two doses of the diluted hexavaccine (Hexacima) supplemented with 14 pmol of the indicated fused or unfused antigens. On day 35, salivation was induced using the parasympathomimetic alkaloid pilocarpine, and saliva was collected. The antibody concentrations were determined by indirect ELISA using specific standard curves for calibration in arbitrary units per milliliter. Bars indicate the geometric mean from two independent experiments run in duplicate. The error bars represent the error of the mean. The dashed lines indicate the cutoff level for the assay defined as the sum of the background concentration of the negative control plus five-times the standard deviation. Kruskal-Wallis test followed by *post-hoc* Dunn´s multiple comparison test was used for statistical analysis. Different letters indicate statistically significant differences.

**Fig 7:**
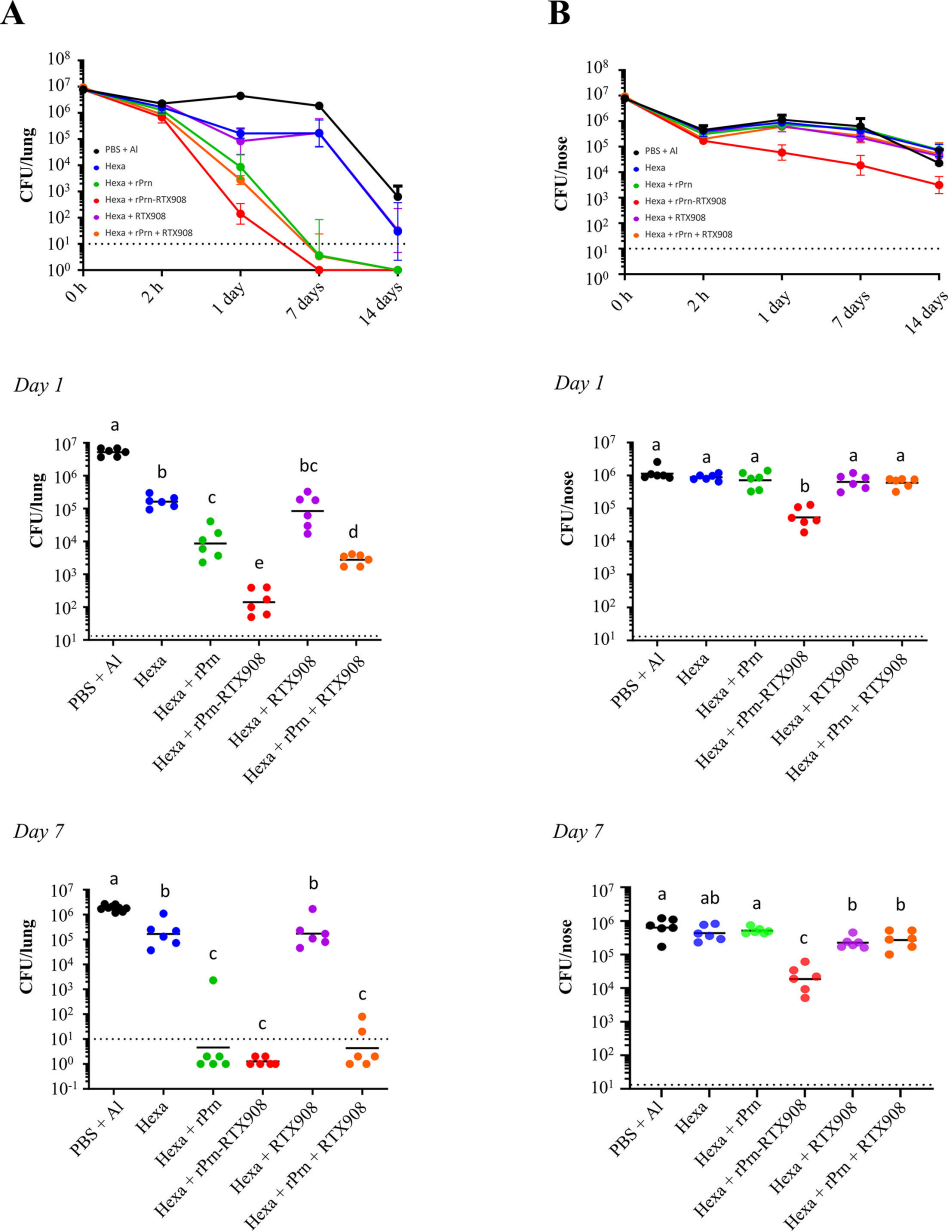
The rPrn-RTX908 fusion antigen is a potent mucosal immunogen that importantly enhances the protective efficacy of an intranasally applied 1/20 reduced dose of the pediatric pertussis vaccine and overrides its blunting effect on nasal cavity protection from *B. pertussis* infection. (A, B) Intranasal immunization with the rPrn-RTX908 fusion antigen admixed into a 1/20 HD pediatric hexavaccine formulation significantly enhances mouse lung (A) and nasal mucosa (B) protection from *B. pertussis* infection. The curves represent the geometric means of bacterial loads (CFU) determined in homogenates of lung and nasal cavity tissues of the intranasally vaccinated and challenged animals (*n* = 6). The panels below show the bacterial loads in the given tissue for each individual animal per experimental group on days 1 and day 7 post-infection. Horizontal bars in the panels indicate the geometric means of CFU per group on days 1 and 7, respectively. The dashed lines indicate the detection limit of the assay. Kruskal-Wallis test and *post- hoc* Dunn’s test were used for multiple comparisons; different letters indicate statistical differences.

Therefore, we examined whether intranasal application of the rPrn-RTX908 antigen in an acellular vaccine formulation could improve the protection of the mouse nasal cavity from *B. pertussis* infection. Toward this aim, we used the licensed alum-adjuvanted pediatric Hexacima vaccine (D-T-aP-Hib-HepB-IPV), the aP component of which only comprises the PT and FHA antigens, but no Prn. Therefore, equimolar (14 pmol) amounts of the alum adjuvanted rPrn-RTX908 antigen, or the rPrn or RTX908 antigens separately or in combination were admixed into the Hexacima vaccine, and the individual formulations were applied intranasally to 6 mice per group in 30 µL of vaccine per mouse (15 µL per nostril, 1/20 human dose (HD) of Hexacima vaccine), using two doses administered at 3 weeks of interval (Fig. 6A). Fourteen days later, blood was sampled by retroorbital punction for determination of antibody levels in serum prior to infection of the vaccinated mice by intranasal application of 1.4 × 10^7^ CFU of *B. pertussis* Tohama I bacteria administered in 20 µL of suspension (10 µL per nostril). The here-used challenge dose was higher by two order of magnitude than the optimal 10^5^ CFU challenge dose used in the lung clearance assay, as the 10^7^ CFU dose has been previously established as an inoculum that allows sufficient resolution of the nasal cavity colonization levels following vaccination (14, 15). Saliva for secretory IgA (sIgA) determination was sampled from parallel groups of immunized animals after pilocarpine induction (53). The intranasal vaccines supplemented with rPrn, RTX908, or rPrn-RTX908 antigens triggered both specific serum IgG responses against the rPrn and RTX908 antigens (Fig. 6B and C), but a well detectable and high-secretory sIgA response in the saliva was induced only by the rPrn-RTX908 fusion antigen and not against the individual-free rPrn or RTX908 antigens, or their mixture (Fig. 6D and E). The serum anti-Prn IgG amounts induced by rPrn alone were comparable to those induced by the rPrn-RTX908 fusion (Fig. 6B), which induced importantly higher levels of anti-RTX specific IgG or sIgA antibodies than the RTX908 antigen alone (Fig. 6C and E). Moreover, the high serum antibody response to intranasal immunization by the recombinant antigens translated into enhanced protection of the vaccinated animals against airway infection by a high inoculation dose (1.4 × 10^7^ CFU) of *B. pertussis* Tohama I bacteria. Compared to mice immunized by the 1/20-HD Hexacima vaccine alone, which yielded clearance of the *B*. p*ertussis* infection from the lungs only past day 7, the mice immunized intranasally by vaccines eliciting also anti-Prn antibodies controlled the lung infection quite efficiently already on day 1 p.i. (Fig. 7A). Moreover, compared to rPrn alone, or to the combination of unfused rPrn and RTX908 antigens jointly administered intranasally in 1/20-HD Hexacima, the rPrn-RTX908 fusion admixed into 1/20-HD Hexacima conferred a significantly higher level of protection, which enabled a near-complete clearance of the infection from the lungs already on day 1 after challenge (Fig. 7A, middle panel). These three groups of animals then cleared the infection from the lungs completely by day 7 p.i., whereas the animals immunized by the 1/20 HD Hexacima vaccine alone still had a rather high bacterial load in the lungs (~10^6^ CFU) (Fig. 7A, lower panel). Moreover, unlike the addition of unfused rPrn alone, or in combination with unfused RTX908, the addition of the rPrn-RTX908 fusion antigen into 1/20 Hexacima yielded an intranasal vaccine that conferred significant protection of the nasal cavity from *B. pertussis* infection (Fig. 7B). Compared to all other groups that exhibited as high bacterial loads in the nasal cavity as the non-immunized control mice over the 14 days after inoculation, the animals immunized intranasally with the rPrn-RTX908 fusion added to a 1/20 HD Hexacima vaccine exhibited importantly lower bacterial loads in the nasal cavity already on day 1 after infection (Fig. 7B, middle panel). This reduced colonization of nasal mucosa by *B. pertussis* was even more pronounced on day 7 p.i., where intranasal immunization with rPrn-RTX908 conferred a decrease of bacterial load by almost two orders of magnitude compared to non-immunized controls and over one order of magnitude compared to animals that received the combination of unfused rPrn and RTX908 antigens in 1/20 HD Hexacima vaccine (Fig. 7B, lower panel).

## DISCUSSION

We show here that a fusion of the antigenic passenger moiety of Prn to the N-terminus of an RTX fragment of the CyaA toxin enables and importantly accelerates folding of the Prn β-solenoid. More importantly, the fusion to RTX908 yields a bi-functional antigen that induces functionally superior antibody responses, eliciting opsonizing anti-Prn, as well as CyaA toxin-neutralizing serum antibodies. Such sera alleviate the block of opsonophagocytic uptake and oxidative burst inhibition imposed on neutrophils by the action of CyaA toxin present at concentrations expected to occur on infected mucosa *in vivo* (30 ng/mL) (48).

The results show that the rPrn-RTX908 fusion is a potent protective antigen when applied intranasally in a mixture with a PT and FHA-containing vaccine (1/20 HD Hexacima). The intranasally administered fusion protein induced strong anti-Prn and anti-CyaA serum IgG and secretory sIgA responses that significantly enhanced the protection of mouse lungs from *B. pertussis* infection. Compared to the PT + FHA vaccine alone, which conferred some clearance of infection from the lungs only beyond day 7 p.i., the intranasally applied vaccine containing the rPrn-RTX908 fusion enabled almost complete clearance of infection from the lungs already on day 1 after infection. The rPrn-RTX908 fusion conferred stronger protection of the lungs than an equimolar amount of the rPrn alone, demonstrating the added value of induction of anti-CyaA antibodies in lung protection from *B. pertussis* infection. Most importantly, unlike the 1/20 HD PT + FHA vaccine containing rPrn or RTX908 antigens individually or in a mixture, the intranasal immunization with the rPrn-RTX908 fusion antigen also induced a strong secretory sIgA response against both rPrn and RTX908 components of the fusion antigen and conferred a significant protection of the nasal cavity from colonization by *B. pertussis*. The simultaneous induction of both serum IgG and of sIgA antibodies against rPrn or RTX908 by the rPrn-RTX908 fusion antigen enabled a reduction of bacterial load in the infected nasal cavity of mice already on day 1 after infection. This resulted in about one and a half of order of magnitude lower bacterial counts in the nasal cavity on day 7 after infection, as compared to mice vaccinated by PT + FHA with rPrn or RTX908, or by a PT + FHA with rPrn + RTX908 mixture. Since the failure to confer protection of the nasal cavity from *B. pertussis* colonization is the major drawback of the current acellular pertussis vaccines (14, 54, 55), these results open intriguing options for improvement of the aP vaccines.

It remains to be investigated why the free rPrn and rsPrn proteins induced several-fold higher anti-Prn serum IgG responses after the 3rd dose of intraperitoneal immunization than the equimolar amounts of the rPrn-RTX908 and rsPrn-RTX908 antigens (c.f. Fig. 4B). At present, we can only speculate that the passenger domain of Prn is a neutral protein, whereas RTX908 a quite acidic protein and hence the free Prn and Prn fused to RTX908 adsorbed to different extent to alum and this may account for the higher immunogenicity of Prn when applied intraperitoneally. Intriguingly, an opposite picture was seen upon intranasal administration of the antigens in a mixture with 1/20 HD of the Hexacima vaccine, where the rPrn-RTX908 fusion antigen triggered much stronger secretory sIgA responses against its rPrn and RTX908 moieties than the unfused antigens admixed individually or in a blend to 1/20 HD of the Hexacima (c.f. Fig. 6D and E). A plausible speculation would be that this was due to enhanced stability of the two antigens when fused together, as indicated by the importantly faster folding of the rPrn antigen in fusion to RTX908 (c.f. Fig. 2). Indeed, the more stable fusion antigen might be more efficiently transported and presented to the B cells in the lymphoid follicles than the free unfused antigens. Moreover, the rPrn-RTX908 fusion protein exhibited an enhanced presentation of the immunodominant conformational epitopes of the flexible R1 and R2 loops of Prn (c.f. Fig. 3). This could possibly be due to locking of the conformation of the R1 and R2 loops in the fusion protein by the C-terminally linked and folded RTX908 moiety, stabilizing the structure of the entire hybrid protein. Intriguingly, previous work of Hijnen et al. (35) indicated a physical interaction of the R1 and R2 loops, as deduced from the observed correlation between the lengths of these immunodominant loops. It is tempting to speculate that when the RTX908 moiety is fused to the C-terminal end of the rPrn protein, its structure is locked in a conformation that exposes protective epitopes of the R1 and R2 loops and enhances their recognition by B cell receptors, thereby enabling induction of protective functional sIgA antibodies capable to block Prn-mediated adhesion of bacteria to epithelial or phagocytic cells. Simultaneously, the stabilization of the fold of the RTX908 moiety by the N-terminally attached rPrn may lead to the induction of functional anti-RTX908 sIgA antibodies that can neutralize the CyaA toxin activity. This would enable effective opsonophagocytic killing and uptake of the bacteria. As a result, faster clearance of the infection from the nasal mucosa would be expected, as observed here for the group of animals intranasally immunized by the rPrn-RTX908 fusion antigen (c.f. Fig. 7B).

In addition to reduction of nasal colonization, as compared to immunization with a mixture of free rPrn and RTX908 antigens, the intranasally applied rPrn-RTX908 fusion protein elicited an enhanced protection of lungs from *B. pertussis* infection, yielding near complete clearance of the bacteria from the lungs by day 1 post-infection. This suggests a functional synergy of the sIgA antibodies induced by the rPrn-RTX908 fusion with the serum IgG responses elicited as well by the intranasal immunization.

It has recently been shown that mobilization of neutrophils especially of the Siglec F^+^ neutrophils onto the infected mucosa of upper airways is crucial for the elimination of *B. pertussis* infection (56). At the same time, the action of the CyaA toxin secreted by *B. pertussis* is notorious for near-instant ablation of bactericidal activities of neutrophils through an increase in cytosolic cAMP levels already at picomolar CyaA toxin concentrations (20, 21, 57). Hijacking of cellular cAMP/PKA signaling by the cell-invasive CyaA toxin was then repeatedly shown to swiftly and potently block the complement-dependent opsonophagocytosis of bacteria and the bactericidal oxidative burst capacity of neutrophils and other CR3-expressing sentinel phagocytes (20, 21, 46, 47, 57). This would go well with the proposed early role of the CyaA toxin in airway colonization (58). Indeed, we show here that the CyaA-neutralizing anti-RTX908 antibodies elicited by the rPrn-RTX908 fusion antigen relieved the block of ROS production and restored the opsonophagocytic uptake of anti-Prn and complement-opsonized *B. pertussis* bacteria by activated dHL-60 neutrophils exposed to 67 pM CyaA toxin (30 ng/mL). This provides a plausible explanation of the enhanced protection of nasal mucosa from *B. pertussis* infection in mice that were intranasally immunized by the rPrn-RTX908 antigen and mounted potent anti-Prn and anti-CyaA serum IgG and secretory sIgA responses (c.f. Fig. 6B through E). This observation complements our early reports that vaccination with the CyaA antigen alone confers some protection of mouse lungs from *B. pertussis* infection (11, 12) and the report of DiVenere and colleagues (16), who showed that passive immunization of mice by intranasal administration of a mixture of 0.5 µg of anti-Prn and 10 µg of CyaA-neutralizing monoclonal antibodies allowed a reduction *B. pertussis* load in infected lungs by 2.5 orders of magnitude at day 3 p.i (16).

The results reported here substantiate the earlier proposition that a CyaA-derived antigen capable of inducing toxin-neutralizing antibodies is a promising candidate for inclusion into the next generation of aP vaccines, as it is expected to enhance their protective capacity. For the sake of simplicity, we used here an alum-adjuvanted vaccine for intranasal application, as this allowed previously to induce potent systemic antibody responses against pertussis antigens in mice (15, 59). Obviously, for a possible human use, an alum-free mucosal vaccine formulation would need to be developed. It is plausible to assume that by using a suitable adjuvant and formulation (60–64), the immunogenicity of the rPrn-RTX908 vaccine antigen candidate could be further enhanced.

## MATERIALS AND METHODS

### Bacterial strains and plasmid constructs

The expression plasmids for the production of the rsPrn and rPrn proteins were constructed by cloning PCR-amplified fragments of the *B. pertussis* Tohama I *prn* gene into the pET28b vector (Novagen). For this, a single forward primer (5′-ATAGCTAGC*GAAAACCTGTATTTTCAGGGC***GACTGGAACAACCAGT** **CCATC**) comprising a NheI restriction site (underlined) and the sequence encoding a TEV protease-cleavage site (italics) was used in combination with two different reverse primers (5′-CGTAAGCTT**TCAGCCGCCGTCGCCGGTG** and 5′-CGTAAGCTT**TCACGCCTTCGCGCCCACCA**) to amplify the *prn* gene fragments encoding the rsPrn and rPrn protein passenger domain proteins, respectively. The fragments were inserted into the pET28b vector using the NheI and HindIII restriction sites, yielding the pET28b-rsPrn and pET28b-rPrn plasmids for production of the rsPrn and rPrn proteins bearing an N-terminal 6×His affinity purification tag. To generate the plasmids for production of the rPrn-RTX908 and rsPrn-RTX908 fusion proteins (c.f. Fig. 1A), the rPrn and rsPrn moiety-encoding DNA fragments were PCR amplified using adapted primer pairs (forward 5′-ATACATATGGACTGGAACAACCAGTCCATC and reverse 5′-CCACCATGGGCCGCCGTCGCCGGTG or 5′-CCACCATGGCGCCTTCGCGCCCACCAG, respectively) and fused in-frame with an RTX908-encoding PCR fragment (forward 5′-ATACCATGGAAACTGGATGTGATCGGCGGA and reverse 5′- CCAGAGCTCGTTGTCCTGG primer) into the NdeI and SacI-digested pT7CT7-RTX908 plasmid (30), yielding the pT7CT7rsPrn-RTX908 and pT7CT7rPrn-RTX908 plasmids that, aside of the rsPrn-RTX908 and rPrn-RTX908 protein fusions, encode also the CyaA modifying protein toxin acyl transferase CyaC (30). All constructs were sequence-verified and complete sequences of the used constructs will be provided upon request.

### Recombinant antigens

The RTX908, rsPrn-RTX908, and rPrn-RTX908 proteins were produced using the pT7CT7-RTX908, pT7CT7-rPrn-RTX908, and pT7CT7-rsPrn-RTX908 plasmids, respectively, into inclusion bodies in *E. coli* BL21/pMM100(*lacI^q^*) cells grown in 1 L of MDO medium (M9 minimal salts, 20 g/L of yeast extract and 20 g/L of glycerol) supplemented with ampicillin (150 µg/mL) and induced with 0.5 mM isopropyl β-D-1-thiogalactopyranoside (IPTG) at *A*_600_ ~0.6 for 4 hours at 37°C with orbital shaking at 140 rpm. Bacterial cells were pelleted at 4,000 × *g* for 35 min at 4°C, washed in 150 mM NaCl, 50 mM Tris-HCl, pH 8, and disrupted by sonication. Inclusion bodies were pelleted at 30,000 × *g* for 30 min at 4°C, washed in the sonication buffer, and dissolved in 8 M urea, 50 mM NaCl, 50 mM Tris-HCl, pH 8. The cleared urea extracts were loaded on DEAE-Sepharose columns equilibrated with 8 M urea, 100 mM NaCl, 50 mM Tris-HCl buffer, and unbound proteins were washed out with 8 M urea, 50 mM NaCl, 50 mM Tris-HCl, pH 8 buffer. The column with bound RTX908 protein and Prn-RTX908 fusions was washed with 10 bed volumes of 6 M urea, 50 mM NaCl, 50 mM Tris-HCl containing 0.5% Triton X-114 at room temperature, followed by 20 bed volumes of detergent-free buffer, to remove LPS (38) and the endotoxin-free proteins were eluted using 8 M urea, 200 mM NaCl, 50 mM Tris-HCl, pH 8, buffer, and stored frozen.

For the production of the free rsPrn and rPrn proteins, inclusion bodies were solubilized with 8 M urea, 50 mM NaCl, and 50 mM Tris-HCl, pH 8. The cleared urea extracts were passed through DEAE Sepharose columns equilibrated with the same buffer to deplete acidic proteins by binding to DEAE-Sepharose under such conditions. The flow-through and wash fractions containing the rsPrn or the rPrn protein (pI > 6.0) were next loaded onto Ni-NTA columns equilibrated in the same buffer (8 M urea, 50 mM NaCl, 50 mM Tris-HCl, pH 8). The columns with bound proteins were washed with 10 bed volumes of 0.5% Triton X-114 in 6 M urea, 50 mM NaCl, 50 mM Tris-HCl, pH 8, to remove LPS and extensively washed with 20 bed volumes of 10 mM imidazole, 8 M urea, 50 mM NaCl, 50 mM Tris-HCl, pH 8, buffer. The proteins were eluted using 50 mM imidazole, 8 M urea, 50 mM NaCl, and 50 mM Tris-HCl, pH 8. The imidazole was removed, and proteins were concentrated by diafiltration with 8 M urea, 50 mM NaCl, 50 mM Tris-HCl, pH 8, buffer on Amicon stirrer cell (Millipore) using a 30 kDa ultracell membrane (Millipore) and stored frozen.

For some experiments, the recombinant antigens were folded prior to use as described by Hijnen et al. (34). Briefly, the 8 M urea-containing stocks of denatured Prn antigens were diluted 17.5-fold by drop-wise addition (0.5 mL/min) with gentle agitation into the urea-free folding buffer 100 mM NaCl, 3 mM CaCl_2_, 200 mM L-Arg, 50 mM Tris-HCl, pH 8, containing the protease inhibitor cocktail (cOmplete EDTA free, SIGMA). The folding process was allowed to complete at 4°C for 24 h without agitation before L-arginine was removed and the proteins were concentrated by ultrafiltration under nitrogen pressure into the same buffer lacking L-arginine using 30 kDa ultracell membrane (cutoff, 30 kDa) and were stored frozen at −20°C until use.

Protein concentrations were determined by spectrophotometry (Denovix DS-11 FX) using the calculated molecular weights and theoretical extinction coefficients and the endotoxin content was determined by a chromogenic LAL endotoxin assay (Charles River Endosave, Charleston, USA).

### Production of native pertactin

Native pertactin was purified from a mutant strain *B. pertussis* Tohama I ΔPT ΔCyaA, not producing the PT and CyaA toxins, by successive ion exchange chromatography on DEAE-Sepharose and SP-Sepharose columns (65). Briefly, the bacteria were grown for 16 h at 37°C with orbital shaking to A_600_ ~2.0 in 2 L of modified SS medium (66, 67) containing 0.5% Heptakis (2,6-di-O-methyl-β-cyclodextrin, Sigma Aldrich CAS: 51166-71-3). Cells were collected by centrifugation at 5,000 × *g* for 30 min at 4°C, washed three times in ice-cold 150 mM NaCl, 50 mM Tris HCl, pH 8, resuspended in the buffer supplemented with cOmplete EDTA-free protease inhibitor cocktail (SIGMA), and heated at 60°C for 1.5 h. The released pertactin was separated from the cells by centrifugation at 5,000 × *g* for 30 min at 4°C, concentrated in 50 mM Tris HCl, pH 7.4, by ultrafiltration, and loaded onto a DEAE-Sepharose column equilibrated in the same buffer at 4°C. Upon wash of the column, the bound pertactin was eluted using 50 mM NaCl in 50 mM Tris HCl. The buffer was exchanged for 100 mM citrate buffer, pH 4, by ultrafiltration, the protein was loaded onto a preequilibrated SP-Sepharose column and LOS was removed by washing with 20 resin bed volumes of citrate buffer containing 0.5% Triton X-114 at 4°C. The detergent was removed by washing the resin bed with 10 column volumes of citrate buffer, pH 4. Pertactin was eluted with citrate (pH 6), transferred into PBS buffer, pH 7.4, containing protease inhibitors, concentrated by ultrafiltration on Amicon 50 mL stirrer cell (Millipore) employing a 30 kDa membrane (Millipore), and stored frozen until use.

### Western blots

The identity of the proteins was confirmed by Western blots using specific antibodies. Briefly, the proteins were separated on 7.5% acrylamide gels, transferred onto PVDF membranes, and detected using a monoclonal antibody directed against a linear epitope of Prn (PeM72, dilution 1/10,000) or by mouse polyclonal serum generated against purified recombinant CyaA (dilution 1/10,000) and revealed by chemiluminescence using an HRP-conjugated anti-mouse IgG antibody (SIGMA) and a SuperSignal West Femto kit (Thermo Scientific).

### Protein folding assessment by fluorimetry

To assess the folding kinetics, the proteins were fast-diluted to a protein concentration of 0.2 mg/mL with 200 mM L-arginine, 3 mM CaCl_2_, 150 mM NaCl, 50 mM Tris-HCl buffer, pH 8, and incubated at 4°C for 0, 5, 24, 48, and 72 h. Fluorescence in the range of 300–400 nm was measured in 3 mm path QS-high precision quartz cells (Hellma Analytics) using an FLS 1000 fluorimeter (Edinburgh Instruments, UK) employing the software Fluoracle 2.4.3. The signal of the buffer was subtracted from the curves. The experiment was repeated two times in duplicate.

### Protein folding assessment by circular dichroism spectroscopy

The folding kinetics of the proteins were monitored by circular dichroism spectroscopy in the function of the changes in the secondary structure content. Due to technical considerations, the dilution buffer was modified to reduce the interferences while keeping the conditions for proper folding of the proteins. The unfolded samples (purified, urea-containing samples) were diluted in 50 mM NaCl, 3 mM CaCl_2_, and 20 mM Tris-HCl, pH 8, to 0.2 mg/mL. Three technical replicates per protein sample were used for measurements. The spectra were collected immediately after dilution and after incubation for 5, 12, or 24 h at 4°C. The CD spectra were obtained using a 0.1 cm path cell (quartz Suprasil 1110-QS, Hellma) in the range of 205–260 nm (Chirascan-plus spectrometer, Applied Photophysics, USA). The data were converted from ellipticity to mean residue ellipticity (deg × cm^2^ × dmol^−1^).

### *Bordetella pertussis* strains and growth conditions

*B. pertussis* Tohama I strain was obtained from the Culture Collection of the Institute Pasteur in Paris under catalog No. CIP 81.32 and cultivated on Bordet-Gengou (BG) agar plates (Difco, USA) supplemented with 1% glycerol and 15% defibrinated sheep blood (LabMediaServis, Czech Republic). A spontaneous Str^R^ *B. pertussis* strain was isolated on BG agar plates supplemented with 1% glycerol, 15% defibrinated sheep blood, and 100 µg/mL streptomycin. The Str^R^ *B. pertussis* strain was stored at −80°C.

Bacterial cultures for intranasal infections of mice were grown for 48 h on nutritionally enriched BG plates containing modified Stainer–Scholte medium (68) supplemented with 3 g/L of Casamino Acids on top of the standard composition. Bacteria harvested from plates were suspended and diluted in prewarmed PBS to the density required for intranasal inoculation.

### Animal immunization and infection

Five-week-old female BALB/cByJ mice (Charles River, France) were immunized by intraperitoneal route with three doses administered 14 days apart, each containing 31.8 pmol of the studied proteins (Fig. 1), adjuvanted with 0.1 mg/dose of InjectTM Alum (stock 13.82 mg Al^3+^/mL) (Thermo Scientific). The control group received only PBS with alum (0.1 mg Al^3+^/dose). Blood was collected from the maxillary vein using an appropriate lancet size on days 0 (preimmune), 14, 28, and 35 to quantify the humoral response elicited after each administration (Fig. 4A). Prior to this sampling, animals were anesthetized by intraperitoneal injection of ketamine (80 mg/kg) and xylazine (8 mg/kg) in 0.9% saline. Serum was obtained by centrifugation at 5,000 × *g* for 10 min at 8°C and stored at −80°C until use.

To assess the protective immunity, vaccinated mice were anesthetized 1 week after the last immunization as described above (Fig. 4A) and intranasally challenged with 1 × 10^5^ CFU of *Bordetella pertussis* Tohama I bacteria administered in a volume of 50 µL. Animals were euthanized on days 0, 3, 5, 7, 14, 21, and 35 p.i. Lungs and nasal cavities with turbinates were aseptically removed and homogenized in physiological saline solution using tissue grinders (Heidolph mechanical stirrers, model RZR 2020, Merck, Darmstadt, Germany) and tissue homogenizer (IKA Ultra turrax T25, Sigma-Aldrich, St. Louis, MO, USA), respectively. Nasal homogenates were cleared by centrifugation at 217 × *g* for 10 min, and dilutions of lung and nasal homogenates were spread on BG agar supplemented with 15% defibrinated sheep blood and 100 µg/mL of streptomycin. CFUs were counted after 4 days of incubation at 37°C. The detection limit of the assay was defined as 20 CFU/mL, representing the lowest bacterial load detectable under our conditions, with organs disrupted in a 2 mL total volume and then 100 µL per sample plated.

For mucosal immunization studies, 1/20 of the human dose (HD) of the commercial formulation of hexavalent pediatric vaccine (Hexacima) containing aP components (chemically inactivated pertussis toxin and filamentous hemagglutinin) was used. The vaccine was supplemented with 14 pmol per dose of rPrn-RTX908, rPrn, and/or RTX908 to generate four different acellular formulations. BALB/cByJ mice (Charles River, France) were sedated by intraperitoneal injection of ketamine (80 mg/kg) and xylazine (8 mg/kg) diluted in a physiological saline solution. Mock immunized animals received only PBS containing the same alum concentration in 1/20-HD of the Hexacima vaccine. The experimental groups of animals received two doses of the corresponding acellular formulation 21 days apart by instillation of 15 µL into each nare (2 × 15 µL per animal). Fourteen days after the second dose, mice were anesthetized, blood was collected by retroorbital puncture and sera were obtained as described above. A parallel group of immunized animals was used to collect pilocarpine-induced saliva for sIgA determination. Under general anesthesia, animals were intraperitoneally injected with 0.5 mg/kg of pilocarpine, a parasympathomimetic alkaloid that promotes salivation (53). Saliva was collected and stored at −20°C till usage.

The immunized animals were then intranasally challenged with 1.4 × 10^7^ CFU of *Bordetella pertussis* Tohama I suspended in 20 µL of PBS. The bacterial loads in lung and nose tissue homogenates were assessed at indicated times as described above. Sera from animals immunized with a commercial whole-cell vaccine (Pentavac SD) were previously obtained by Holubova et al. (14).

### Antibody quantitation and avidity assessment

NUNC Maxisorp plates were coated with 3 µg/mL of native pertactin or RTX908 in 3 mM CaCl_2_, 100 mM NaCl, 50 mM Tris HCl, pH 7.4, overnight at 4°C. The wells were washed with 3 mM CaCl_2_, 100 mM NaCl, 0.05% Tween 20, 50 mM Tris HCl, pH 7.4, and blocked with the dilution buffer (1% BSA, 0.05% Tween 20, 3 mM CaCl_2_, 100 mM NaCl, and 50 mM Tris HCl, pH 7.4) before diluted sera were added for 90 min at RT. An anti-mouse IgG conjugated to horseradish peroxidase (HRPO) was used as the secondary antibody (SIGMA) and the signal was developed using the substrate solution TMB/H_2_O_2_. The absorbances were measured at 450 nm with a reference wavelength of 630 nm using a Tecan Spark instrument (Männedorf, Switzerland). A hyperimmune anti-CyaA serum was employed to construct the anti-RTX908 standard curve, which was calibrated in arbitrary units per milliliter (AU/mL). For the construction of the anti-Prn standard curve, the monoclonal antibody PeM72 was employed. The IgG concentrations were calculated using a four parameters logistic equation tool of the software MyAssays Online (https://www.myassays.com).

sIgA concentrations in pilocarpine-induced saliva were determined using the same ELISA protocol, with anti-IgA-HRPO (Abcam) as the secondary antibody. The standard curve was calibrated in arbitrary units per milliliter (AU/mL). The detection limit was defined as the blank signal plus five times its standard deviation, while the positivity threshold was defined as the signal of the negative control plus three times its standard deviation.

To assess the avidity of the specific antibodies, a modified ELISA including a step of incubation with ammonium thiocyanate was employed. The sera were diluted to a concentration yielding an ELISA signal (absorbance at 450 nm [*A*_450_]) of 0.9–1.2. The avidity index (AI) was defined as the percentage of the *A*_450_ signal of the wells treated with dilution buffer containing 0 or 1.5 mM ammonium thiocyanate, divided by the signal of untreated wells. The samples were run in duplicate and evaluated in three independent experiments.

### Epitope presentation assessment by inhibition ELISA

To assess the relative antigenicity of the proteins, an inhibition ELISA assay was performed (scheme in Fig. 3B) using the previously described anti-Prn monoclonal antibodies PeM1, PeM2, PeM29, and PeM72 (39) that were kindly provided by Dr. Guy Berbers (RIVM Bilthoven, The Netherlands). NUNC Maxisorp plates were coated overnight at 4°C. Suitable working concentrations of the used anti-Prn mAbs were determined in trial experiments and antibody titration reactions with individual antigens were kept overnight at 4°C in non-adsorbing microtiter plates. The formed antigen-mAb complexes were transferred into the antigen-coated wells of ELISA plates for binding inhibition equilibria establishment over 90 min at room temperature. The plates were washed and the relative amounts of bound mAbs were detected as described above. The inhibition index was calculated as follows: inhibition index = 100 - (100 × OD_*x* nmol_/OD_0 nmol_), where OD_*x* nmol_ was the absorbance at concentration *x* of the inhibitor protein and OD_0 nmol_ was the signal in the absence of the competitor.

### CyaA neutralization assay

Human monocytic THP-1 cells (ATTC number TIB-202) were cultured at 37°C under a humidified 5% CO_2_ atmosphere in RPMI 1640 (Sigma, USA) medium supplemented with 10% (vol/vol) fetal calf serum (GIBCO Invitrogen, USA) and Antibiotic Antimycotic Solution (Sigma, USA). CyaA toxin (1 µg/mL) was pre-incubated with 1:50 diluted mouse sera at 4°C for 30 min and added to 10^6^ /mL THP-1 cells in D-MEM medium (Sigma, USA). The toxin was allowed to bind cells at 4°C for 30 min before the cells were extensively washed and subsequently lysed with 0.1% Triton X-100 to determine the cell-associated adenylate cyclase (AC) enzyme activity in the presence of 1 µM calmodulin as previously described (69). One unit of AC activity corresponds to 1 µmol of cAMP per minute at 30°C (pH 8.0). CyaA binding was expressed as the percentage of toxin binding to THP-1 cells, where 100% corresponds to CyaA binding in the presence of sera from mock-immunized mice.

### Opsonophagocytosis uptake assay

HL-60 cells were differentiated with DMF diluted in D-MEM medium supplemented with 20% FCS, 0.03% pyruvate, and 0.01% L-glutamine. mScarlet-expressing *Bordetella pertussis* Tohama I (14) was grown on Bordet-Gengou blood agar plates and resuspended to OD 1. The bacteria were seeded into U bottom well plates and incubated with heat-inactivated serum (dilution 1:10) and baby rabbit serum serving as complement source (dilution 1:10) for 15 min at 37°C. CyaA was added to the corresponding wells at a final concentration of 30 ng/mL. The dHL-60 cells were seeded into the wells to reach an MOI of 20:1. The plate was centrifuged for 10 min at 600 × *g* to facilitate the contact between the cells and the bacteria (41). Hanks’ buffer with 2 mM CaCl_2_, 2 mM MgCl_2_ , and 1% glucose was used for all the dilutions/suspensions. The plate was incubated for an additional 45 min at 37°C and then the cells were extensively washed with cold PBS. After a blocking step with 5% BSA, the surface CD11b/CD18 receptor was stained using a specific antibody (clone OKM1) conjugated to Dyomics 488. The cells were washed three times with cold PBS to reduce unspecific binding. The association of mScarlet-expressing *Bordetella pertussis* bacteria with HL-60 cells was analyzed by flow cytometry using a Becton Dickinson LSRII instrument, the data were processed using FlowJo, and the graphs were constructed using GraphPad Prism software. The results were expressed as the percentage of opsonophagocytic uptake. The mScarlet-expressing *Bordetella pertussis* uptake in the absence of CyaA was taken as 100%.

### ROS production determination

ROS production was assessed as previously described by Cerny et al. (21) using dHL-60 cells loaded with 150 µM luminol for 15 min at 37°C. The bacteria were opsonized with the sera and the complement source as described above, using flat-bottom white plates. CyaA was diluted with Hanks’ buffer (supplemented with 2 mM CaCl_2_, 2 mM MgCl_2_, and 1% glucose), and added to the corresponding wells. The cells were seeded into the wells and the luminescence was continuously measured every 1.5 min for 3 h at 37°C in a Tecan Spark device. The area under the curve (AUC) for the treatments in the presence and absence of CyaA were calculated, and the data were expressed as the percentage of ROS production, where the AUC of corresponding treatments in the absence of CyaA was taken as 100%.

### Statistical analysis

Statistical parameters (central tendency, dispersion, and specialized averages) were calculated using Microsoft Excel and GraphPad Prism 10 software. For multiple comparisons across experimental groups, the non-parametric Kruskal-Wallis test and the *post hoc* Dunn’s multiple comparison test were employed. The non-parametric Mann-Whitney *U* test was used when the comparison of two independent experimental groups was needed.

## Data Availability

Data are available at https://doi.org/10.5281/zenodo.14892995. More data will be made available on request.

## SUPPLEMENTAL MATERIAL

Supplemental Figures msphere.00959-24-s0001.pdf Figures S1 to S6.

ASM does not own the copyrights to Supplemental Material that may be linked to, or accessed through, an article. The authors have granted ASM a non-exclusive, world-wide license to publish the Supplemental Material files. Please contact the corresponding author directly for reuse.
