# Supplementary material for: Intranasal application of a bifunctional pertactin-RTX fusion antigen elicits protection of mouse airway mucosa against Bordetella pertussis colonization
Source: mSphere. 2025 Mar 31;10(4):e00959-24. doi: 10.1128/msphere.00959-24 (PMC12039270; doi:10.1128/msphere.00959-24)
Supplement: Supplemental Figures — Figures S1 to S6. [file msphere.00959-24-s0001.pdf]

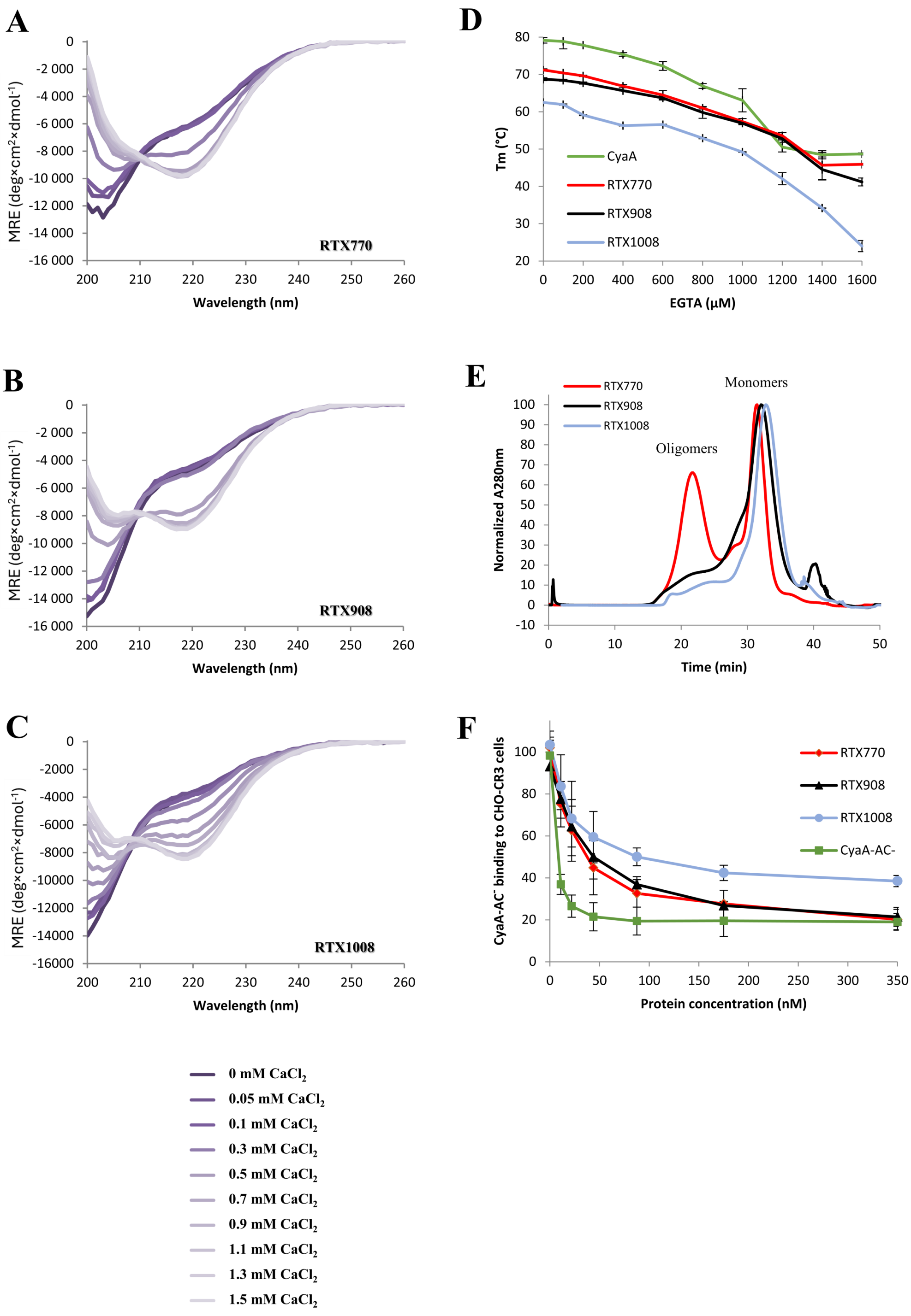


**Figure S1.** Characterization the CyaA-derived constructs RTX770, RTX908 and RTX1008 (30). **A-C)** Folding of the constructs monitored by circular dichroism. The preparations were diluted >60-fold from 8 M containing stocks into 50 mM NaCl, 20 mM Tris HCl pH 8 to a protein concentration of 0.2 mg/ml and kept at room temperature before loaded with increasing concentrations of $Ca^{2+}$ ions (added as calcium chloride) from 0-1.5 mM. The spectra were collected using a quartz 0.1 cm path -length cuvette. The graphs correspond to the average of the mean residue ellipticities (MRE) calculated for the proteins RTX770 (**A**), RTX908 (**B**) and RTX1008 (**C**). **D)** Thermal stability of the proteins evaluated by nanoscale differential scanning fluorimetry. The proteins were diluted into an urea-free buffer containing 1.5 mM $CaCl_2$, 50 mM NaCl, 20 mM Tris HCl pH 8 and titrated with increasing concentrations of EGTA. The fluorescence signals at 350 nm and 330 nm were recorded in the interval of temperatures from 25 to 90 °C and the melting temperatures (Tm) were calculated from the melting curves. The graph shows the average of three technical replicates. The error bars indicate the standard deviation. **E)** Folding and aggregation tendency of the proteins RTX770, RTX908 and RTX1008. The urea-unfolded proteins were injected into a Superdex 200 10/300 column preequilibrated with 3 mM $CaCl_2$, 150 mM NaCl, 50 mM Tris HCl pH 8 and folded on column, and elution was monitored as absorbance at 280 nm. **F)** Competition for CR3 receptor biding with CyaA. $1\times10^5$ transfected CHO cells expressing the CR3 receptor heterodimer CD11b/CD18 (17) were incubated with increasing concentrations of the tested proteins in D-MEM medium containing 1.9 mM $Ca^{2+}$ on ice for 15 min. Fluorescently labelled CyaA-AC$^-$ conjugated to Dyomics 647 was added to a final concentration of 1 μg/ml and the competition for CR3 binding equilibrium was allowed to establish for 30 min on ice before binding of the Dyomics 647-CyaA-AC$^-$ to cells was determined by flow cytometry. The populations of dead/live cells were analyzed using Hoechst 33258. The graph shows the means of data from three experiments with standard deviations.

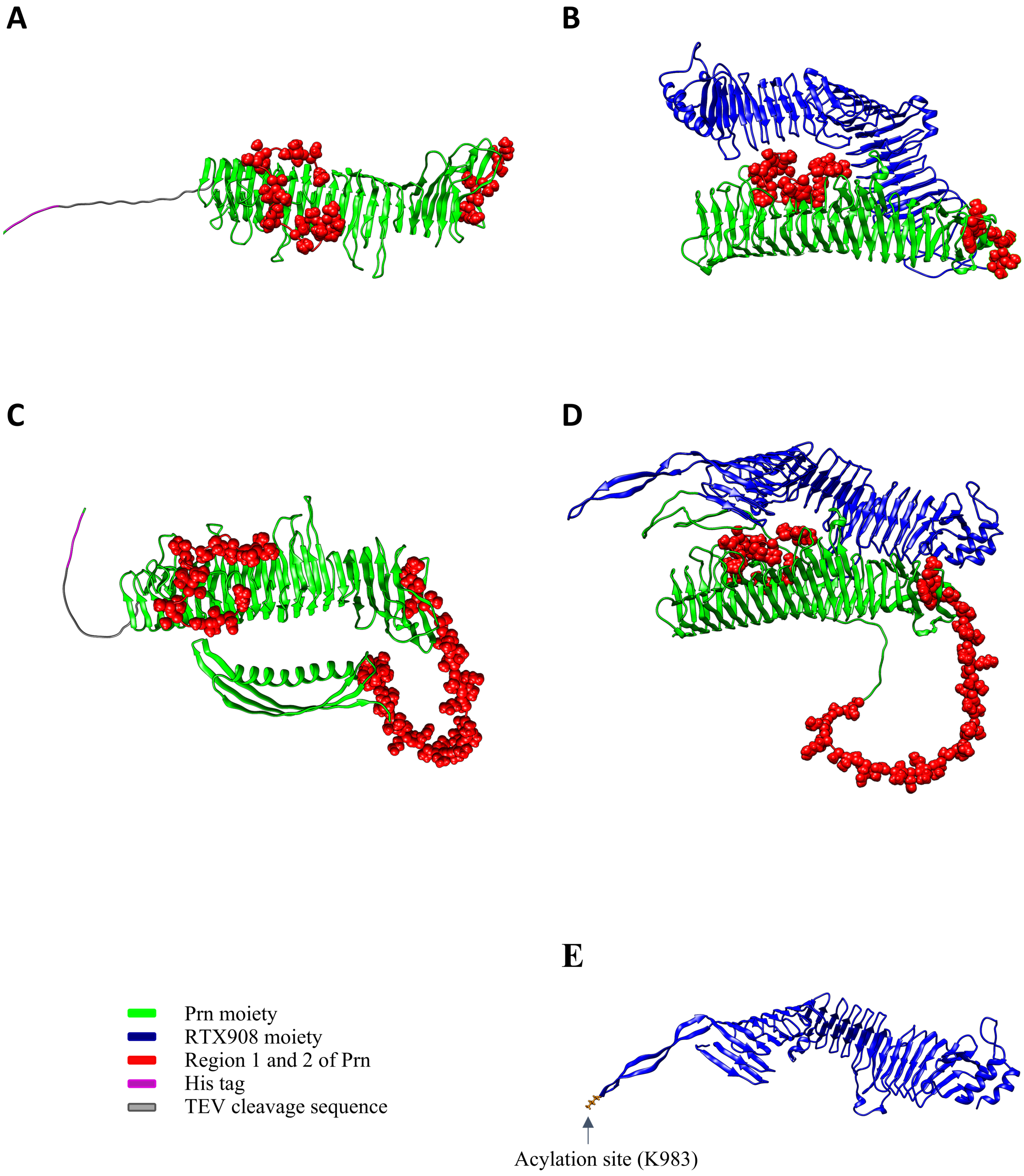


**Figure S2.** Alphafold prediction (38) of the tertiary structures of the studied proteins. rsPrn (**A**), rsPrn-RTX908 (**B**), rPrn (**C**), rPrn-RTX908 (**D**) and RTX908 (**E**). The loops of Region 1 and Region 2 of Prn are highlighted as balls model for better visualization. The side chain of the lysine residue at position 983 of CyaA sequence is highlighted by an arrow in the figure **E**.

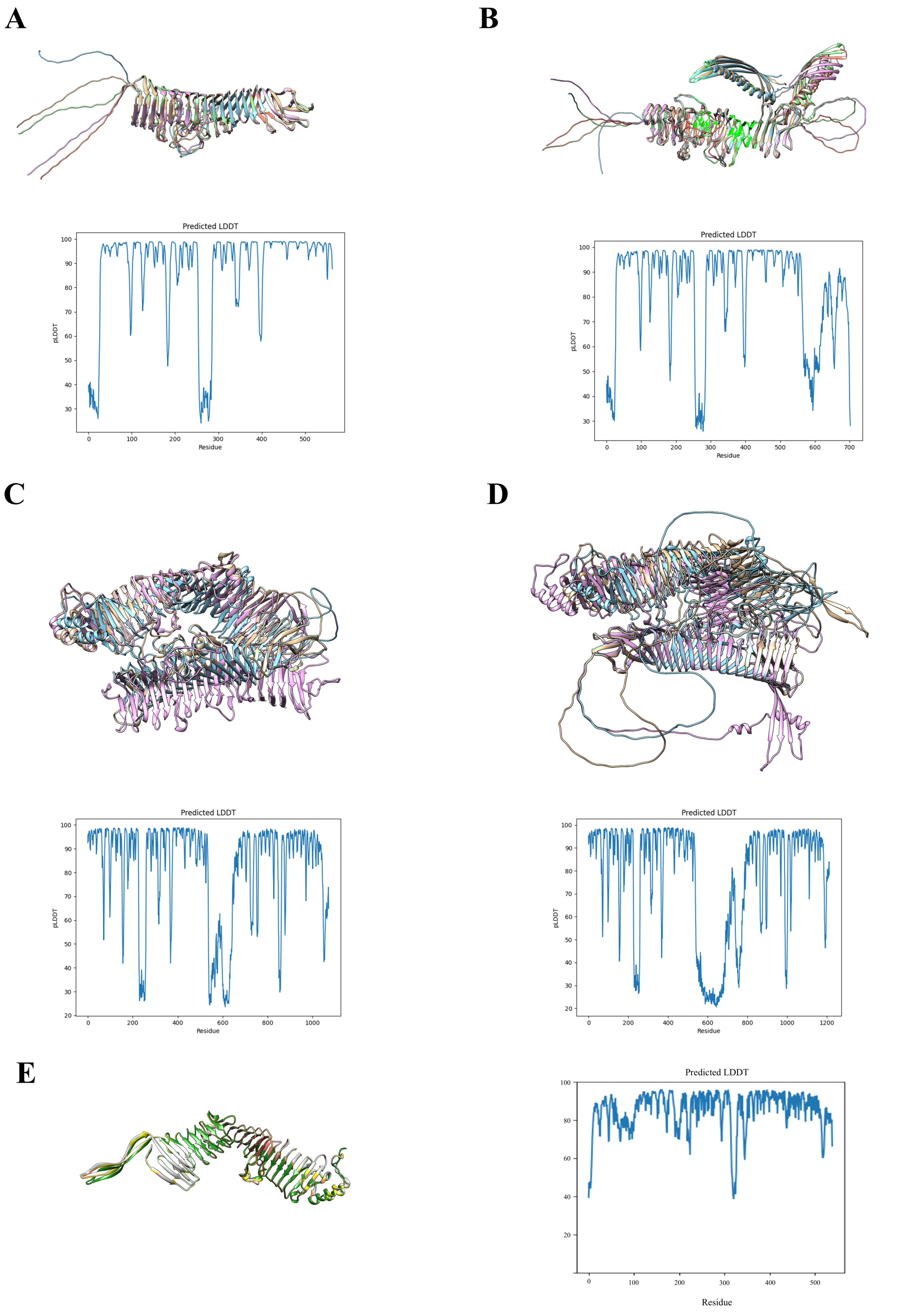


**Figure S3.** Alphafold models (38) of the tested proteins. The figures present the superposition of 5 models predicted by the Alphafold algorithm with the corresponding LDDT (local distance difference test) plots for the proteins rsPrn (**A**), rPrn (**B**), rsPrn-RTX908 (**C**), rPrn-RTX908 (**D**) and RTX908 (**E**).

**A**

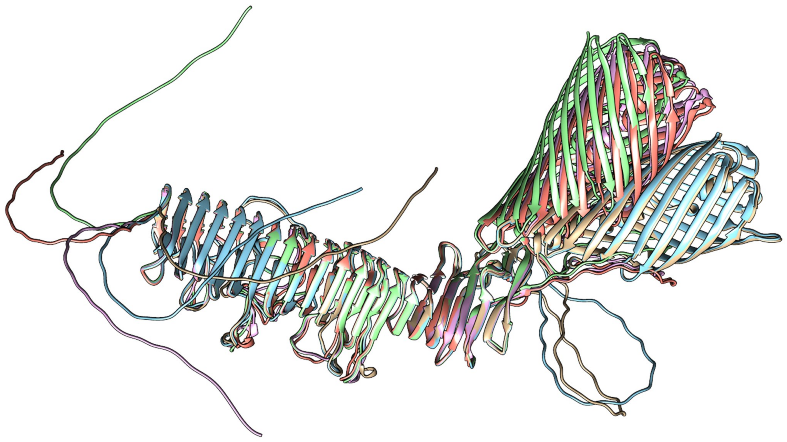

**B**

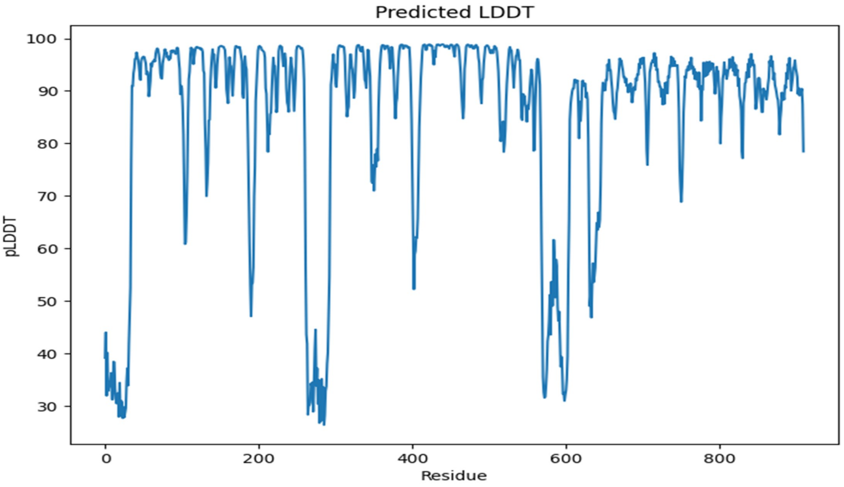


**Figure S4.** Predicted structure of unprocessed Prn P.93. **A**) Superposition of the five models predicted for the full-length Prn protein by Alphafold based on the sequence of the gene encoding for the pertactin autotransporter P.93. The processed N-terminal signal peptide was included in the models. **B**) LDDT (local distance difference test) plot calculated by Alphafold (38).

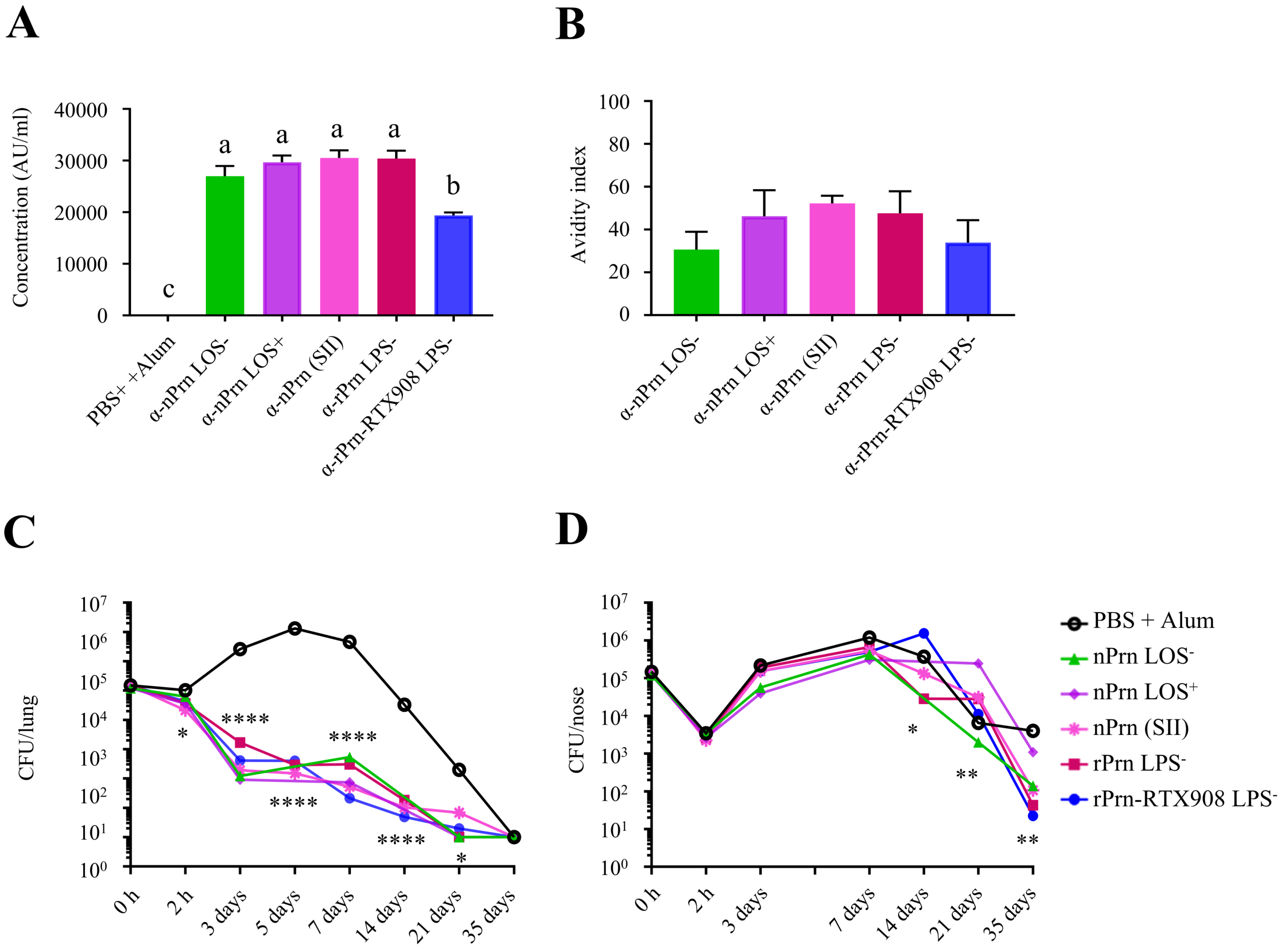


**Figure S5. A)** Immunogenicity of native and recombinant Prn preparations prior and after LOS (*B. pertussis*) / LPS (*E. coli*) depletion. BALB/cByJ mice were immunized intraperitoneally with three doses of the alum-adjuvanted Prn protein preparations on days 0, 14 and 28. Blood was sampled on day 35 by retroorbital puncture. The concentrations of total serum anti-Prn IgG were quantified by indirect ELISA using a standard curve established with the monoclonal antibody PeM72. Kruskal Wallis test, *post hoc* Dunn's multiple comparison test. Different letters indicate statistical differences. **B)** Avidity of the anti-Prn antibodies determined by indirect ELISA. Anti-Prn IgG from appropriately diluted immune sera ($A_{450} = 1$) was bound to plate wells coated with nPrn and incubated for 90 min at room temperature. The wells were washed repeatedly, and sodium thiocyanate was added to a 1.5 M concentration for 15 min. The plates were washed again, and the amounts of residual bound IgG were quantified by ELISA. The avidity index was calculated using the ratio of the absorbance signals without (0 M) or with (1.5 M) thiocyanate treatment ($OD_{1.5\ mM}/OD_{0\ mM}$). The values represent the means of three independent experiments run in duplicate with error bars for standard deviation. Low (AI ≤ 30%), Medium (30% < AI > 60%), High (AI ≤ 60%). **C-D)** Immune protection elicited by Prn containing various level of LOS/LPS contamination. BALB/cByJ mice were intraperitoneally immunized three times with 31.8 pmol doses of the indicated immunogens adjuvanted with alum at two weeks intervals. Seven days after the last vaccine dose the animals were intranasally infected with $1\times10^5$ CFU of *Bordetella pertussis* WT bacteria administered in 20 µl of suspension (10 µl per nostril). Groups of mice were sacrificed at indicated time points after infection and bacterial loads in the lungs (**C**) and nasal cavities (**D**) of infected animals were determined upon plating of lung and nasal tissue homogenates on Bordet-Gengou blood agar plates for CFU counting after 5 days of plate incubation at 37 °C. Data points represent the geometric means of CFU from 3 to 9 infected animals per time point. Kruskal Wallis test; all the experimental groups were compared with the mock group (PBS + Al). *, p<0.05; **, p<0.01; ***, p<0.001; ****, p<0.0001.

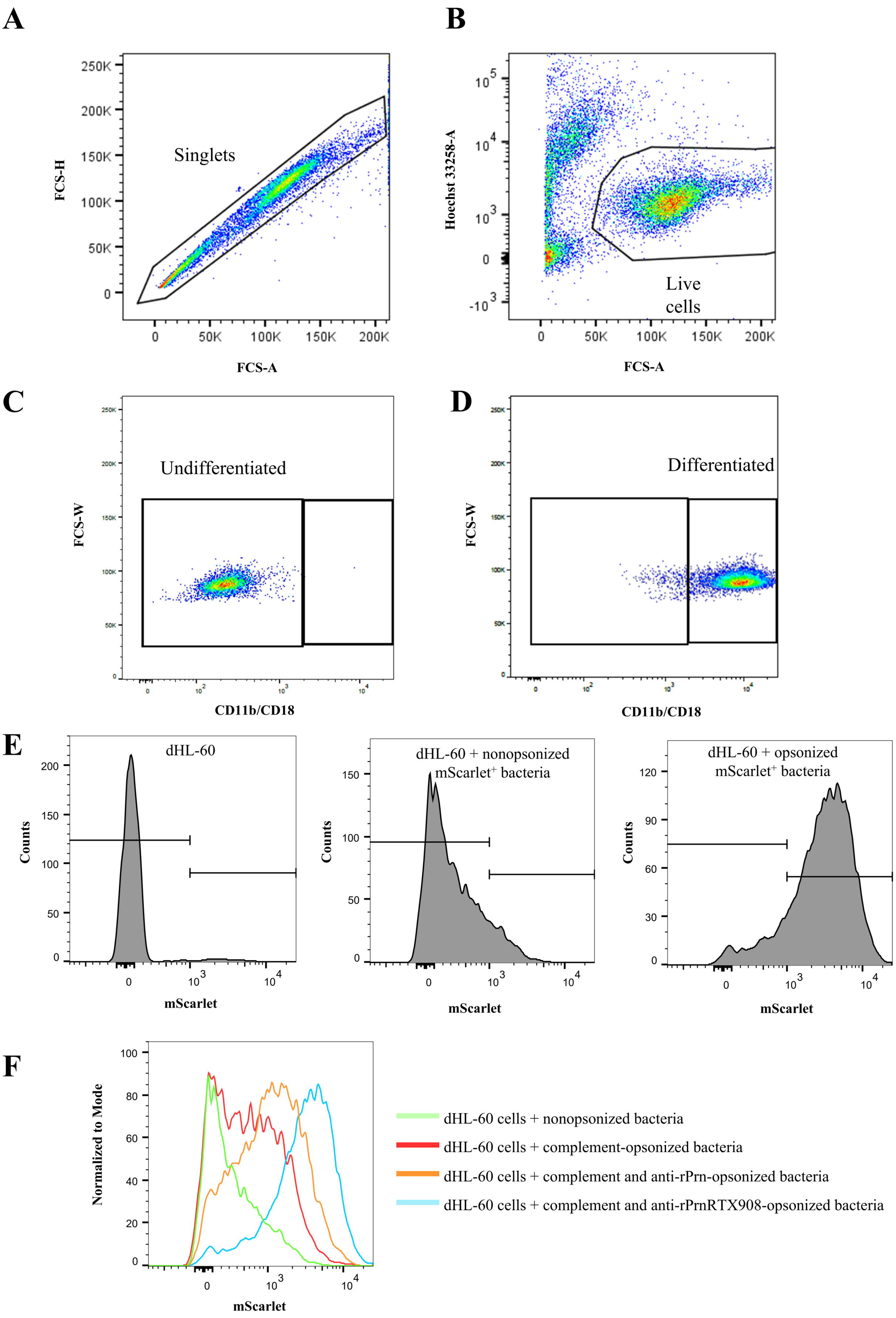


**Figure S6.** Gating strategy used for opsonophagocytosis assay evaluation. The singlets were selected using the density plot of FCS-A versus FSC-H (**A**). From this population, the live cells were gated (**B**) to discard dead cells and debris. Undifferentiated HL-60 cells were used to set the boundaries of CD11b/CD18 expression between the undifferentiated (**C**) and differentiated (dHL-60) cells (**D**). **E)** Histograms of counts versus mScarlet fluorescence intensity. From the left to the right: dHL-60 cells, dHL-60 cells with nonopsonized mScarlet-expressing bacteria and dHL-60 cells incubated with bacteria opsonized with complement and immune serum. **F)** Comparison of histograms of mScarlet fluorescence of dHL-60 cells with nonopsonized or opsonized fluorescent bacteria.
